# Postmortem examination of COVID‐19 patients reveals diffuse alveolar damage with severe capillary congestion and variegated findings in lungs and other organs suggesting vascular dysfunction

**DOI:** 10.1111/his.14134

**Published:** 2020-07-05

**Authors:** Thomas Menter, Jasmin D Haslbauer, Ronny Nienhold, Spasenija Savic, Helmut Hopfer, Nikolaus Deigendesch, Stephan Frank, Daniel Turek, Niels Willi, Hans Pargger, Stefano Bassetti, Joerg D Leuppi, Gieri Cathomas, Markus Tolnay, Kirsten D Mertz, Alexandar Tzankov

**Affiliations:** ^1^ Pathology Institute of Medical Genetics and Pathology University Hospital Basel University of Basel Basel Switzerland; ^2^ Institute of Pathology Cantonal Hospital Baselland Liestal Switzerland; ^3^ Intensive Care Unit University Hospital Basel University of Basel Basel Switzerland; ^4^ Division of Internal Medicine University Hospital Basel University of Basel Basel Switzerland; ^5^ University Department of Medicine Cantonal Hospital Baselland Liestal Switzerland

**Keywords:** autopsy, cardiovascular, lung, SARS‐CoV‐2, COVID‐19, senile amyloidosis, kidney

## Abstract

**Aims:**

Coronavirus disease 2019 (COVID‐19), caused by severe acute respiratory syndrome coronavirus 2 (SARS‐CoV‐2), has rapidly evolved into a sweeping pandemic. Its major manifestation is in the respiratory tract, and the general extent of organ involvement and the microscopic changes in the lungs remain insufficiently characterised. Autopsies are essential to elucidate COVID‐19‐associated organ alterations.

**Methods and results:**

This article reports the autopsy findings of 21 COVID‐19 patients hospitalised at the University Hospital Basel and at the Cantonal Hospital Baselland, Switzerland. An *in‐corpore* technique was performed to ensure optimal staff safety. The primary cause of death was respiratory failure with exudative diffuse alveolar damage and massive capillary congestion, often accompanied by microthrombi despite anticoagulation. Ten cases showed superimposed bronchopneumonia. Further findings included pulmonary embolism (*n* = 4), alveolar haemorrhage (*n* = 3), and vasculitis (*n* = 1). Pathologies in other organ systems were predominantly attributable to shock; three patients showed signs of generalised and five of pulmonary thrombotic microangiopathy. Six patients were diagnosed with senile cardiac amyloidosis upon autopsy. Most patients suffered from one or more comorbidities (hypertension, obesity, cardiovascular diseases, and diabetes mellitus). Additionally, there was an overall predominance of males and individuals with blood group A (81% and 65%, respectively). All relevant histological slides are linked as open‐source scans in supplementary files.

**Conclusions:**

This study provides an overview of postmortem findings in COVID‐19 cases, implying that hypertensive, elderly, obese, male individuals with severe cardiovascular comorbidities as well as those with blood group A may have a lower threshold of tolerance for COVID‐19. This provides a pathophysiological explanation for higher mortality rates among these patients.

## Introduction

Coronaviruses are enveloped, positive single‐stranded RNA viruses, which predominantly cause upper respiratory tract infections,^1^ and are also associated with gastroenteritis and necrotising enterocolitis in children.^2^ Previous outbreaks caused by coronaviruses include severe acute respiratory syndrome (SARS) in 2003^3^ and Middle East respiratory syndrome (MERS) in 2012.^4^ Both coronaviruses originated from bats, which infected secondary hosts such as civet cats (SARS) and dromedary camels (MERS).^5^ Early cases of coronavirus disease 2019 (COVID‐19) were first described in late 2019, when a series of previously unidentified pneumonia‐related deaths emerged in Hubei province, China. COVID‐19, caused by SARS coronavirus 2 (SARS‐CoV‐2), subsequently spread to> 205 countries and territories, with a current count of> 2.5 million cases and> 170 000 deaths worldwide.^6^ Clinical data suggest that SARS, MERS and COVID‐19 manifest with similar respiratory symptoms of varying severity.

Since the outbreak, healthcare professionals and researchers have been working together to decipher the pathophysiology of COVID‐19. Although the latest literature provides insights into clinical manifestations,^7^ published histopathology and autopsy findings currently remain scarce.^8^, ^9^, ^10^, ^11^, ^12^, ^13^

Several studies presenting postmortem findings of SARS patients have been published, but only few of them incorporated whole body autopsies.^14^, ^15^ Although SARS coronavirus (SARS‐CoV) was detected in many organ systems, the primary finding associated with the cause of death was respiratory failure due to diffuse alveolar damage (DAD). Bronchopneumonia was also a common finding, especially in patients with longer disease duration, who then also showed signs of organising pneumonia.^16^, ^17^ In some cases, secondary lymphoid organs showed profound lymphoid depletion and disruption of architecture, indicating a complex pathophysiology beyond the respiratory system. Instead, like other generalised viral infections, such as human immunodeficiency virus infection, SARS affects the immune system, purportedly leading to generalised immune system dysfunction.^18^ This might be a major difference between infections caused by SARS‐CoV and SARS‐CoV‐2, the latter being more contagious but less virulent.^19^

Autopsy studies on MERS are limited; according to our knowledge, there is currently only one complete autopsy case study described in the literature.^20^ A 45‐year‐old previously healthy male with typical clinical manifestations of MERS died 8 days after first hospital admission. Similarly to what is seen with SARS, major histomorphological findings included exudative DAD, type II pneumocyte hyperplasia, single syncytial cells, and interstitial septal lymphoid infiltrates. The trachea and bronchi also showed lymphoid infiltrates. The kidneys and the heart predominantly showed changes related to hypertension. The spleen and lymph nodes showed an increase in immunoblasts and reactive changes, whereas left‐shifted myelopoiesis was seen in the bone marrow. Ancillary studies using immunohistochemistry and electron microscopy revealed the exclusive presence of MERS coronavirus (MERS‐CoV) in the lungs and submucosal bronchial glands, and confirmed dipeptidyl peptidase 4, a cell surface receptor, as a point of entry for MERS‐CoV. This led to the conclusion that the pathological findings in other organs were probably attributable to secondary causes (due to previous steroid medication) and sequelae of shock. A postmortem investigation of core needle biopsies yielded similar results but did show viral particles in the kidneys.^21^

Owing to our comparatively high autopsy rate (~20% of hospital deaths in the last decade), we have the opportunity to rely on a well‐equipped and experienced team for autopsy‐related investigations. Our study summarises the findings of a comprehensively analysed autopsy cohort of 21 patients from two Swiss hospitals in order to provide an overview of organ pathologies related to COVID‐19.

## Materials and methods

### Study Cohort

All autopsies were performed at the Institute of Pathology at the University Hospital of Basel, Switzerland (*n* = 11) and the Institute of Pathology at the Cantonal Hospital Baselland, Switzerland (*n* = 10). In most cases, full body autopsy was performed (*n* = 17). Partial autopsy of the upper respiratory tract, lungs and heart was carried out in some cases, owing to the wishes of patients or relatives, or excessive obesity. Clinical features, including comorbidities, radiological findings, and medication history, are shown in Table 1 and Table S1. The mean postmortem interval from death to autopsy was 33.3 h (11–84.5 h). This study received approval from the Ethics Committee of Northwestern and Central Switzerland (ID 2020‐00629).

**Table 1:** Summary of clinical parameters

Parameter	Value
Sex: male/female ratio	17:4
Age (years), mean (range)	76 (53–96)
Days in hospital (range)	5.7 (0–16)
Number (%) of intubated patients	6 (30)
Hours between death and autopsy (range)	33.2 (11.0–84.5)
Comorbidities, *n* (%)	
Hypertension	21 (100)
Cardiovascular disease	15 (71)
Smoker	8 (40)
Pre‐obesity/obesity (WHO grade 1/2/3)	10/1/1/4 (48/5/5/19)
Diabetes mellitus	7 (35)
Chronic neurological condition	5 (24)
Chronic obstructive pulmonary disease	3 (15)
Malignancy	3 (15)
Chronic liver disease	2 (10)
Chronic kidney disease	4 (19)
Acquired immunosuppression	1 (5)
Initial clinical presentation, *n* (%)	
Cough	16 (76)
Fever	12 (57)
Dyspnoea/tachypnoea	10 (48)
Pancytopenia	2 (10)
Diarrhoea	1 (5)
Acute or acute‐on‐chronic kidney injury	12/18 (67)
Radiological findings, *n* (%)	
Ground glass infiltrates	12 (57)
Initial laboratory findings upon admission*	
Haemoglobin (120–180 g/l), level (range)	122.4 (97–209)
Anaemia, *n* (%)	7/11 (64)
Total white blood cell count (3.5–10 × 10^–9^/l), value (range)	8.7 (3.3–24.7)
Leucopoenia, *n* (%)	3/11 (27)
Leucocytosis, *n* (%)	3/11 (27)
Neutrophils (40–74%), % (range)	84 (63.4–96.4)
Lymphocytes (19–48%), % (range)	9.4 (1.0–23.5)
Platelets (150–450 × 10^–9^/l), value (range)	229.4 (25–433)
Creatinine (μmol/l), level (range)	254.7 (39–623)
ASAT (11–34 U/l) (in 10 patients), level (range)	67.2 (22–214)
LDH (<135 U/l) (in 10 patients), level (range)	450.5 (171–661)
D‐dimers (<0.19 μg/ml) (in 5 patients), level (range)	4.0 (0.4–10.4)
IL‐6 (<7 ng/l) (in 5 patients), level (range)	217.6 (103–278)
Intake of agents interfering with RAAS^†^, *n* (%)	14/21 (67)
Intake of anticoagulation^*^, ^‡^, *n* (%)	11/11 (100)

ASAT, aspartate aminotransferase; IL, interleukin; LDH, lactate dehydrogenase; RAAS, renin–angiotensin–aldosterone system; WHO, World Health Organization.

All normal ranges of laboratory values with designated units are shown in parentheses.

* Basel cohort only.

^†^ Agents interfering directly or indirectly with the RAAS: angiotensin‐converting enzyme inhibitors, angiotensin II receptor blockers, and aldosterone inhibitors.

^‡^ Agents included heparin and its derivatives, new oral anticoagulants (NOACs), warfarin, and vitamin K antagonists.

### Autopsy Technique Applied

As a safety precaution against infection, our institute developed a COVID‐19‐optimised autopsy protocol in line with recently published recommendations.^22^ Two hours prior to autopsy, 4% phosphate‐buffered formalin was instilled into the mouth, nose, and pharynx. Autopsies were performed in a room with adequate airflow (more than six air changes per hour of total room volume) under conditions similar to those recommended for autopsies of those who have died from suspected Creutzfeld–Jakob disease (i.e. hazmat suits, boots, goggles, and FFP2/3 masks) with an *in‐corpore* technique analogous to that used in forensic institutions. Thoracic organs were eviscerated (see below), and the heart was separately dissected in the direction of blood flow. Parenchymal organs (liver, spleen, kidney, and pancreas) were dissected within the body. After mobilisation of the small and large intestines, their exterior surfaces were inspected; if there were any outstanding findings, tissue was excised for further macroscopic and histological examination. In all cases, tissue samples from the liver, heart and kidney were histologically examined. Other organs, as well as bone marrow and the brain, were analysed upon specific clinical indication (younger age; neurological symptoms). To avoid aerosolisation, the brain was removed by opening the skull with a handsaw; bone marrow from the iliac crest was also procured with a handsaw. A detailed description of this *in‐corpore* protocol is provided in Doc. S1.

The lungs, trachea and larynx were exenterated intact and perfused via the trachea with 4% refrigerated (4°C) phosphate‐buffered formalin. The trachea was then closed with a clamp, and the specimens immersed in formalin at room temperature for 72 h before dissection. The lungs were subsequently cut into 5–10‐mm parasagittal slices and examined macroscopically. Two sections of each lobe, as well as the trachea, were submitted for histological examination.

### Ancillary Techniques

Tissue samples were processed with standard histochemical methods, i.e. haematoxylin and eosin staining (all organs), chromotrope aniline blue staining (heart and liver), Giemsa staining (bone marrow), and periodic acid–Schiff reaction (bone marrow, kidney, and spleen). Additional stains [Congo red, Prussian blue and rhodamine (copper), Gram, Brown–Brenn and/or Grocott methenamine silver stain] were used when necessary.

Immunohistochemical examinations were performed for fibrin, amyloid transthyretin (ATTR), CD3, CD4, CD8, CD20, CD68, multiple myeloma 1 and thyroid transcription factor 1 (TTF1) as previously described.^23^, ^24^, ^25^

In all cases, the postmortem viral load was measured in lungs and other selected organs by means of a quantitative reverse transcription polymerase chain reaction (RT‐qPCR) assay. RNA from formalin‐fixed paraffin‐embedded tissue was extracted with the RecoverAll Total Nucleic Acid Isolation Kit (Thermo Fisher Scientific, Waltham, MA, USA). Viral genomes were detected with the TaqMan 2019‐nCoV Assay Kit v1 (Thermo Fisher Scientific), which targets three different viral genomic regions (ORFab1, S protein, and N protein) and the human RNase P gene (*RPPH1*). The number of viral genomes was determined with the TaqMan 2019‐nCoV Control Kit v1 (Thermo Fisher Scientific) and a comparative *C*_т_ (ΔΔ*C*_т_) method. The method generates individual copy numbers for human *RPPH1* and the three SARS‐CoV‐2 targets. Mean copy numbers of SARS‐CoV‐2 targets were scaled to 1 × 10^6^ *RPPH1* copies.

Electron microscopy was performed on two lung and kidney specimens according to standard institute protocols. Tissue was fixed in 3% glutaraldehyde and examined by use of a transmission electron microscope (Morgagni 268D; FEI Company, Hillsboro, OR, USA).

## Results

### Clinical Characteristics

Our patient cohort had a male/female ratio of 17:4 (i.e. 81% males), and a mean age of 76 years (53–96 years). All patients were diagnosed with COVID‐19 by use of an antemortem polymerase chain reaction (PCR) test for SARS‐CoV‐2 performed on a nasopharyngeal swab, bronchoalveolar lavage fluid, or sputum (median of 7.15 days before death; range, 0–20 days). Most commonly, patients presented with dry cough and fever (*n* = 16 and *n* = 12, respectively). The mean hospitalisation time before death was 5.7 days (range, 0–16 days). One patient died in the emergency room prior to admission to the ward. Six patients had been intubated.

All patients suffered from comorbidities, with hypertension and (pre‐)obesity being most common. Fourteen patients (67%) had a history of renin–angiotensin–aldosterone system (RAAS)‐modulating drug intake. Two patients had been treated with immunosuppressive drugs before contracting COVID‐19, and one suffered from acquired immunodeficiency. In this cohort, 65% had blood group A—although the population incidence in Switzerland is 45%,^26^ this discrepancy did not reach statistical significance.

Clinical characteristics, symptoms and radiological and laboratory findings are summarised in Table 1. Detailed information is given in Table S1.

### Lung and Upper Airway Findings

**Figure 1:**
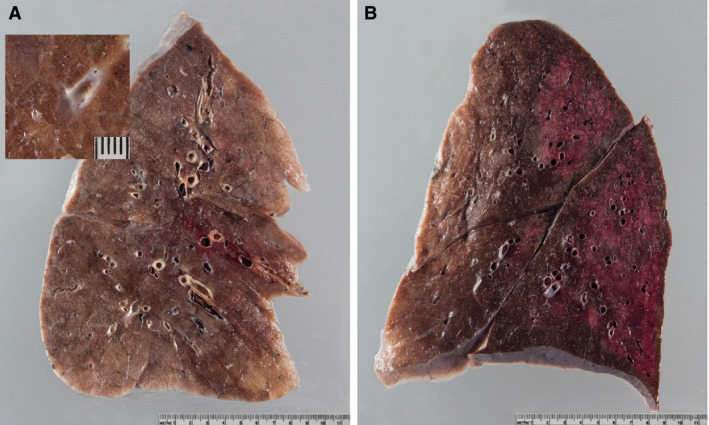
Gross lung findings. **A**, Typical appearance of coronavirus disease 2019 (COVID‐19) lungs; note the perceptibly thickened alveolar septae and congestive interstitial aspects and a thrombembolus in the lower lobe. Insert: detailed view highlighting interstitial congestion. **B**, Extensive bronchopneumonic infiltrates in a COVID‐19 patient suffering from superimposed suppurative pneumonia.

**Figure 2:**
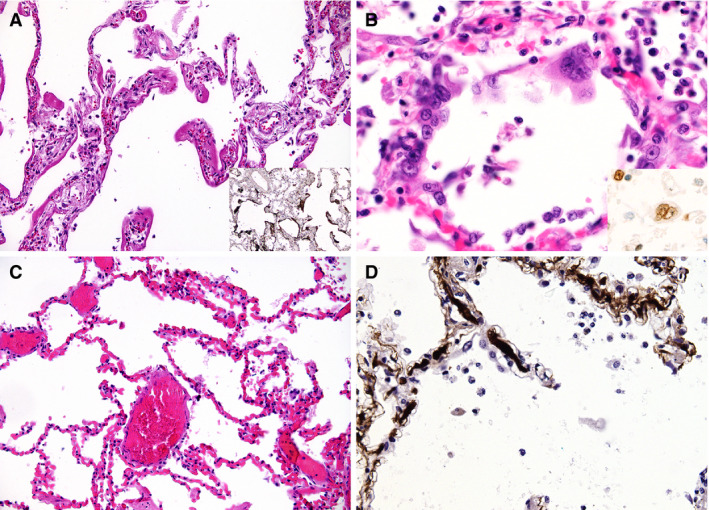
Microscopic lung findings. **A**, Exudative diffuse alveolar damage (DAD) showing discrete hyaline membranes and prominent capillary congestion [haematoxylin and eosin (H&E)]. Insert: immunohistochemistry (IHC) for fibrin(ogen) showing the extent of hyaline membranes. **B**, Syncytial cells of pneumocyte II origin (H&E). Insert: IHC for thyroid transcription factor 1. **C**, Extensive capillary congestion without DAD (H&E). **D**, Microthrombi in alveolar capillaries (IHC for fibrin).

One‐third of patients presented with severe mucous tracheitis/tracheobronchitis. Gross findings in the lungs were heterogeneous (Figure 1A,B), ranging from patchy to diffuse areas of consolidation to severe and extensive suppurative bronchopneumonic infiltrates. In all cases, the lung parenchyma was heavy and firm, and unevenly bluish–red in colour, with signs of severe congestion. The most prominent histological finding was severe capillary congestion (capillarostasis) accompanied by hyaline membranes, reactive pneumocyte changes and syncytial cells corresponding to exudative DAD (Figure 2A). Eight cases showed proliferative DAD (38%). In 10 cases, superimposed bronchopneumonia with both a focal and a diffuse (n = 6) distribution was seen. On immunohistochemistry, syncytial cells were shown to be of pneumocytic origin (expression of TTF1; Figure 2B). Most lungs showed a paucity of interstitial leucocytes, especially lymphocytes, whereas three showed a moderate lymphoid inflammatory infiltrate; in one of these three cases, no viral RNA was detectable in the lungs as determined by postmortem PCR. Three cases showed severe and extensive bronchopneumonia without typical features of DAD; however, in areas without pneumonia, severe capillarostasis was consistently noted (Figure 2C). Other lung pathologies included oedema and alveolar haemorrhage in conjunction with pulmonary embolism. One case, additionally suffering from bronchopneumonia, showed focal vasculitis and capillaritis with a predominantly neutrophilic infiltrate. In five of 11 cases in which immunohistochemistry for fibrin was performed, microthrombi were detected in alveolar capillaries (Figure 2D). Four cases showed peripheral and prominent central pulmonary embolism. Table 2 summarises the most important autopsy findings. Detailed autopsy findings for each patient are shown in Table S2. Scanned slides of each patient showing representative findings in the lungs, heart and kidney, as well as other relevant organs, are available in Doc. S2.

**Table 2:** Summary of autopsy findings

Organ	Diagnosis	*n*	%
Lung	Pulmonary capillary congestion	21/21	100
	DAD, exudative	16/21	76
	DAD, proliferative	8/21	38
	Reactive pneumocytes and syncytial cells	11/21	52
	Microthrombi of alveolar capillaries	5/11	45
	Bronchopneumonia, diffuse	6/21	29
	Bronchopneumonia, focal	4/21	19
	Severe mucous tracheitis	6/21	29
	Emphysema	6/21	29
	Pulmonary embolism	4/21	19
	Prominent lymphoid infiltrates	3/21	14
	Pulmonary haemorrhage	3/21	14
	Amyloidosis of pulmonary vessels	3/21	14
	Vasculitis	1/21	5
Heart	Myocardial hypertrophy	15/21	71
	Senile amyloidosis	6/21	29
	Peracute myocardial cell necrosis	3/21	14
	Acute myocardial infarction	1/21	5
Kidney	Acute tubular damage	14/15	93
	Disseminated intravascular coagulation	3/17	18
	Hypertensive nephropathy	2/17	12
	Diabetic nephropathy	2/17	12
Liver	Steatosis	7/17	41
	Shock necrosis	5/17	29
	ASH/NASH	3/17	18
Lymph node	Increased presence of plasmablasts	5/9	56
	Congestion	6/9	67
Spleen	Acute splenitis	6/21	29
Bone marrow	Reactive left shift of myelopoiesis	3/5	60
	Involvement by haematopoietic malignancies	2/5	40

ASH, alcoholic steatohepatitis; DAD, diffuse alveolar damage; NASH, non‐alcoholic steatohepatitis.

Transmission electron microscopy was performed on two cases. Unfortunately, subcellular structures could not be analysed, owing to autolysis. However, fibrin precipitates were detected within alveolar capillaries in both cases.

### Cardiovascular System Findings

Myocardial hypertrophy was a common finding (71%), correlating with the high prevalence of hypertension in this cohort. There was one case with acute myocardial infarction. Peracute focal necrosis of cardiomyocytes as a sequela of shock was seen in three patients. Six patients aged 76–96 years were diagnosed with senile cardiac amyloidosis, as confirmed by immunohistochemistry for ATTR upon autopsy. Three of these cases showed amyloid deposits in pulmonary vessels. The prevalence of senile amyloidosis in our cohort achieved statistical significance when compared with age‐matched autopsies performed at our institute in 2018–2019 (six of 21 versus 22 of 345 autopsies, *P* = 0.000137, Mann–Whitney *U*‐test). Severe generalised atherosclerosis, defined by the presence of ulcerated plaques of the aorta, was present in 70% of cases.

### Kidney Findings

**Figure 3:**
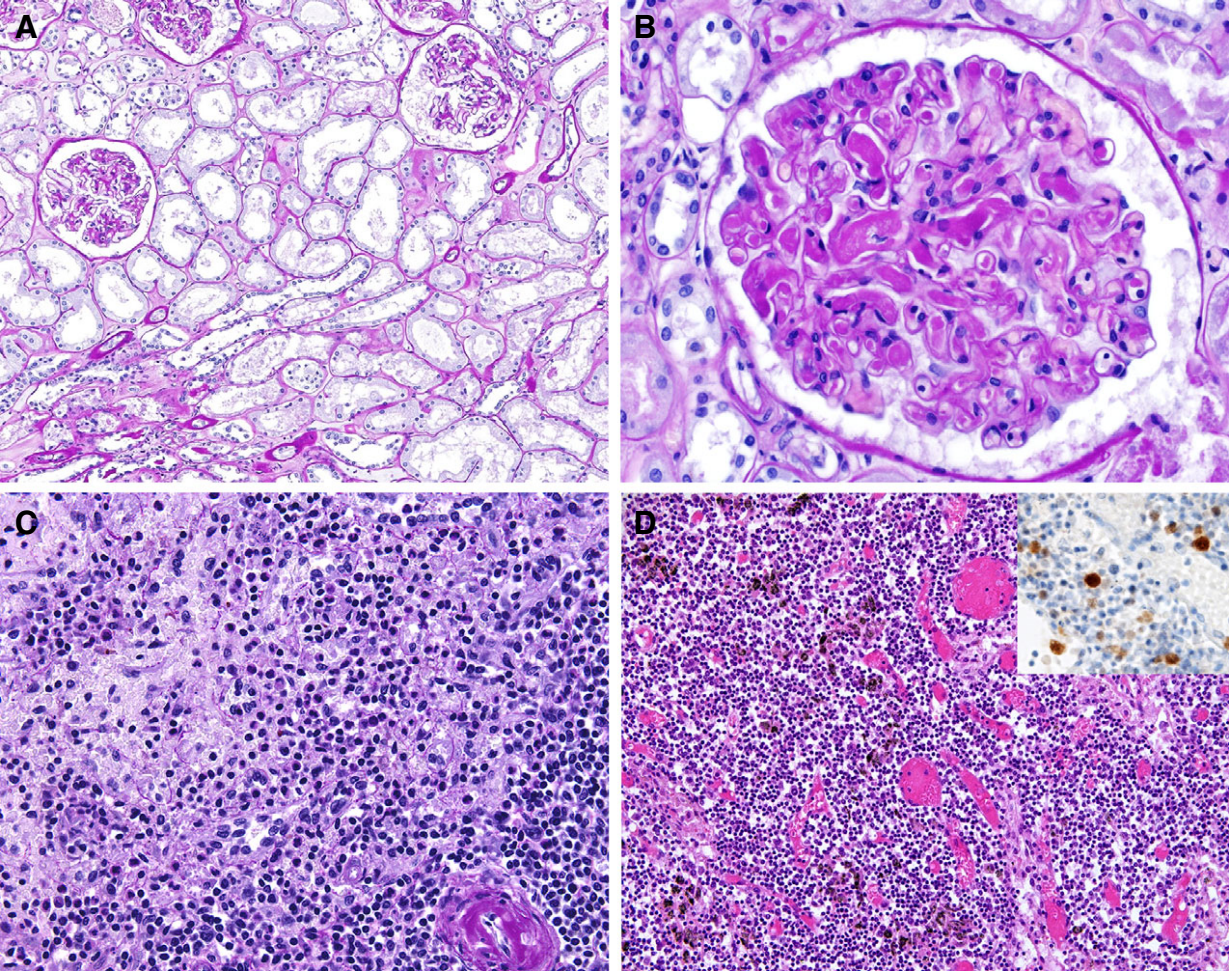
Findings in other organs. **A**, Kidney showing acute tubular damage without evidence of increased inflammatory infiltrates [periodic acid–Schiff (PAS) stain]. **B**, Kidney showing disseminated intravascular coagulation (PAS). **C**, Florid splenitis showing increases in neutrophil numbers in the perifollicular and marginal zones of the spleen (PAS). **D**, Lymph node showing an increase in the number of plasmablasts in the interfollicular zone as well as congestion (haematoxylin and eosin). Insert: immunohistochemistry for multiple myeloma 1.

On gross examination, renal signs of shock were seen in most autopsies. Histologically, these findings correspond to diffuse acute tubular injury with widened tubular lumina, flattened tubular epithelium, and interstitial oedema (Figure 3A). Three of 18 patients showed signs of disseminated intravascular coagulation, with small fibrin thrombi in glomerular capillaries (Figure 3B). One of these cases also presented with an anaemic infarct. Thrombi in other vessels or vasculitic changes were not seen. A focal and sparse chronic inflammatory infiltrate was present in a few cases in areas with interstitial fibrosis and tubular atrophy. Pre‐existing chronic changes, such as arteriolosclerosis, intimal fibrosis of arteries, and vascular scarring related to hypertension and/or ageing, were present in the majority of cases. These findings were particularly prominent in the four patients with known chronic kidney disease of either diabetic or hypertensive aetiology.

**Figure 4:**
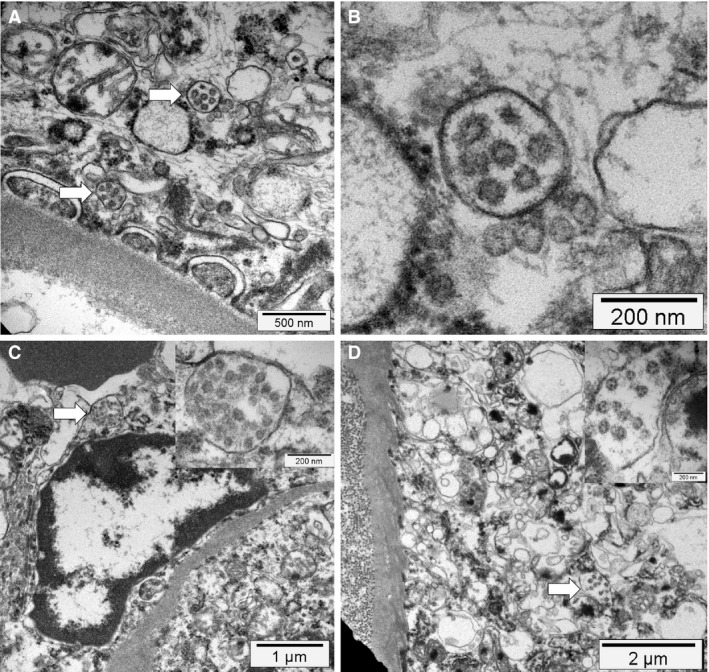
Electron microscopy findings. **A**,**B**, Podocyte cytoplasm with its foot processes on top of a glomerular basement membrane containing mitochondria (upper left corner) and multiple vesicles, two of which contain several small possible virus‐like particles with sizes between 70 nm and 110 nm (arrow). At higher magnification, the vesicles contain double membranes and the virus‐like particles show a ring of electron‐dense granules and a ragged outer contour (electron microscopy). **C**, An activated glomerular endothelial cell, and a vesicle close to the luminal border with virus‐like particles (arrow and insert), adjacent to an erythrocyte (electron‐dense structure at the top left) (electron microscopy). **D**, Cytoplasm of a proximal tubular epithelial cell on top of a basement membrane and adjacent collagen fibres (left side). The cytoplasm contains mitochondria, rough endoplasmic reticulum, and multiple vesicles, one of which contains virus‐like particles (arrow and insert, electron microscopy).

Transmission electron microscopy was performed on two cases with a short (<12 h) postmortem period. In both cases, we observed prominent activation of podocytes, endothelial cells and proximal tubular epithelial cells. The cytoplasm of podocytes contained multiple vesicles, some with attached ribosomes and double membranes. Occasionally, virus‐like particles (7–110 nm) with electron‐dense granules were detected within these vesicles (Figure 4A,B). Sporadically, these particles were present in endothelial cells (Figure 4C) and proximal tubular epithelial cells (Figure 4D).

### Shock Sequelae and Findings in Other Organs

Signs of shock constituted a common finding, predominantly affecting the liver and adrenal gland.

Among bone marrow samples, three of four showed reactive left‐shifted myelopoiesis and one case showed prominent hyperplasia of cytotoxic CD8‐positive T cells. In cases with bronchopneumonia, acute splenitis and/or septic neutrophilic leucocytosis of the red pulp was seen (Figure 3C). Perihilar and paratracheal lymph nodes showed prominent congestion and sinus dilatation, as well as an increase in the number of reactive plasmablasts (Figure 3D), consistent with an activated immune response. Extensive depletion of lymphoid cells as described in SARS was not seen.

Microscopic analysis of the brain revealed no inflammatory infiltrates or neuronal necrosis. Three of four brains examined showed mild hypoxic injury.

### Detection of the Virus According to PCR and Central Nervous System Findings

In all but one patient (see above), mean 125 000 (range 16 865‐749 402) viral copies/1 × 10^6^ *RPPH1* copies were detected in lung tissue by the use of SARS‐CoV2‐specific RT‐qPCR. In other organs (brain, heart, testicle, and kidney), variable, predominantly low, RNA copy numbers were detected. In the brain, copy numbers were generally low, although values in the olfactory bulb were higher than those in the brainstem.

## Discussion

Our study presents a comprehensively analysed autopsy series of predominantly Caucasian COVID‐19 patients. To the best of our knowledge, this cohort is larger than any autopsy cohort of COVID‐19, SARS or MERS patients reported so far. In general, our cohort was older (average age 76 years) than previously published SARS and MERS autopsy collectives. Morphological manifestations in the lungs and other organ systems in COVID‐19 patients were not as extensive and severe as in both SARS and MERS patients.^16^, ^17^, ^18^, ^19^, ^20^, ^21^ This fits with epidemiological studies comparing different types of coronavirus.^19^ Comorbidities in our cohort correspond with clinical observational studies.^27^, ^28^ Other interesting characteristics of our group included a striking predominance of males and a higher incidence of blood group A.

To minimise autolysis, bodies were promptly stored at 4°C after death, and the period between death and autopsy was kept as short as possible (median of 32.9 h). By applying an *in‐corpore* autopsy technique and performing formalin fixation of thoracic organs, we strove to optimise staff safety while taking recently published recommendations into account.^22^ None of the staff members involved in this study developed COVID‐19.

Similarly to other coronaviruses, SARS‐CoV‐2 replicates in the cytoplasm and is assembled within vesicles prior to its release without evoking typical viropathic changes.^29^, ^30^ The upper respiratory tract and lungs serve as the predominant sites of entry and replication,^31^ but other replication sites, such as endothelial cells^32^ and the kidney, have been suggested.^33^, ^34^ As expected, major morphological findings pertaining to the cause of death of our cohort were localised in the respiratory tract.^7^ There was some discrepancy from the clinical diagnoses, as cases clinically diagnosed as ‘pneumonia, COVID‐related’ only showed signs of DAD and showed no signs of suppurative bronchopneumonia, both macroscopically and histologically. Direct correlation with radiology findings was not possible, as most cases had only undergone computed tomography imaging at the time of hospital admission, and not in the course of their stay. The predominant histopathological findings in the lungs were capillary congestion, microthrombi, and, in 48% of cases, moderate intra‐alveolar fibrin exudation corresponding to exudative DAD and superimposed bronchopneumonia. We considered bronchopneumonia, both in an acute and in an organising state, to be the result of bacterial superinfection, and not a direct result of SARS‐CoV‐2‐induced lung tissue damage. Similarly to what is seen in SARS^14^, ^15^ and MERS^20^ patients, syncytial cells originating from type II pneumocytes were observed and there was a paucity of inflammatory infiltrates—especially lymphoid cells—in cases without concomitant bronchopneumonia. DAD was less pronounced in the patients with microthrombi, as also shown by Magro *et al*.,^13^ and, additionally, four patients developed acute pulmonary embolism. Although the presence of microthrombi can be a feature of DAD, we deduce from the former that these findings may, rather, represent a histological correlate of coagulopathies described in COVID‐19 patients.^35^ They corroborate the above‐mentioned observation and are consistent with complement‐mediated microvascular injury in the lung and/or skin.^13^ One patient with superimposed bronchopneumonia showed florid vasculitis of small veins and capillaritis, which has previously been described in individual SARS cases.^14^, ^15^ However, vasculitic changes of other organs or clinical findings related to systemic vasculitis were not seen, implying that the vasculitic changes could have developed in conjunction with severe bronchopneumonia, in contrast to a study that detected systemic vasculitis in COVID‐19 patients.^32^

A novel and unexpected finding of our study linking a fatal COVID‐19 disease course with vascular dysfunction^36^ was a high incidence of ATTR amyloidosis (*n* = 6/21; all diagnosed at autopsy), which was more than four times more prevalent than in non‐COVID‐19 autopsies conducted at our institutes during previous years. In line with this and with cumulating epidemiological evidence, hypertensive, elderly, overweight and male patients, who are particularly known for their hypercoagulability,^37^ were overly represented in our cohort.

Our series contains a high number of patients who concomitantly presented with acute‐onset or acute‐on‐chronic‐onset renal failure (12 of 18 patients at admission; 14 of 18 during the disease course), corroborating previously reported observations of a poorer outcome among this patient group.^38^ In all cases with clinical evidence of kidney failure, acute tubular injury was the main histological finding, in line with a recently published report.^33^ However, this histological finding is unspecific, and multiple aetiologies relate to it. Three of 18 cases investigated contained microthrombi in glomerular capillaries, which are typically observed in the context of disseminated intravascular coagulation and are usually attributable to generalised shock. However, renal SARS‐CoV‐2 replication could potentially have contributed to the acute kidney injury in our cohort. The following observations support this hypothesis: (i) high amounts of viral RNA were detected in kidney samples; (ii) the two samples studied with transmission electron microscopy showed virus‐like particles in podocytes, glomerular endothelial cells, and especially in proximal tubular epithelial cells; and (iii) some of the patients developed microthrombi in spite of timely anticoagulation. In contrast to the study mentioned above,^33^ we detected virus‐like particles within vesicles and not in the cytoplasm, consistent with the coronavirus replication cycle and ultrastructural studies of infected cell cultures.^29^, ^30^

A higher incidence of blood group A among COVID‐19 patients in a large population study from China^39^ correlates with the incidence in our cohort. Evidence suggests that blood group A may be associated with the failure of the pulmonary microcirculation and coagulopathies in COVID‐19 patients.^35^ ABO alleles genetically determine/increase von Willebrand factor (VWF) activity by ⁓20%.^40^ Individuals with the A1A1 genotype in particular show the highest blood group antigen loading of VWF,^41^ thus leading to higher risk and severity (odds ratio 2.6) of thrombosis in the venous system.^42^, ^43^ Moreover, evidence from previous investigations of SARS‐CoV suggests a direct interaction between blood group antigen A and the viral S protein, thus facilitating virus entry via angiotensin‐converting enzyme (ACE) 2,^44^ which was postulated to have a direct effect on the number of infected individuals and disease kinetics in SARS.

Two‐thirds of our cases presented with a history of antihypertensive therapy intake directly or indirectly affecting the RAAS, such as ACE inhibitors, angiotensin II type 1 receptor blockers (ARBs) or aldosterone antagonists. These agents are among the antihypertensive drugs most frequently used in Switzerland. Previous studies have demonstrated that SARS‐CoV‐2 utilises ACE2 as a receptor for viral cell entry.^45^ As ACE2 is up‐regulated upon the administration of antihypertensive therapy affecting the RAAS, these agents might increase the susceptibility to viral invasion, thus increasing the risk of severe and lethal disease outcomes.^46^ However, other experimental evidence has shown a protective effect of RAAS inhibition in COVID‐19 patients,^47^ and, in a recent retrospective clinical study, the use of ACE inhibitors and ARBs was associated with lower overall mortality among hospitalised COVID‐19 patients with hypertension.^48^

All of the points above and a recent autopsy observation of endothelial damage in the kidneys and intestines in COVID‐19 patients^32^ thus strongly suggest the importance of virus‐induced vascular dysfunction in disease progression. Notably, suppressing a COVID‐19‐associated ‘cytokine storm’^49^ with anti‐interleukin‐6 therapy is now a pivotal part of COVID‐19 treatment.^50^ Current investigations are focusing on the complement system^13^ and its role in endothelial dysfunction and coagulopathy; eculizumab, a monoclonal antibody directed against complement protein C5,^51^ has shown promise in SARS‐CoV and MERS‐CoV animal models,^52^ and its efficacy is currently being investigated in a clinical trial.^53^ Given the evidence of endothelial dysfunction in COVID‐19 patients, the efficacy of therapeutic measures such as plasma infusion and exchange and beyond may be considered.^54^

Several investigations have suggested neuronal manifestations of SARS‐CoV‐2, ranging from smell and taste dysfunction to central dysregulation of breathing.^55^ In our series, four brains were analysed histologically, and no inflammatory infiltrates or neuronal necroses were observed. Interestingly, although the viral load was low in all samples, it was slightly higher in the olfactory bulb than in the brainstem, supporting the hypothesis of viral entry into the brain via the lamina cribrosa.

In summary, our findings provide an insight into the complexity of COVID‐19 pathophysiology. SARS‐CoV‐2 substantially contributed to fatality in all cases, but we postulate a multifactorial cause of death, with COVID‐19 as a contributory factor in multimorbid patients. Major findings that imply an impaired microcirculation include pulmonary capillarostasis and the presence of microthrombi in the lungs and kidneys despite anticoagulation. Our findings corroborate clinical and epidemiological data on cardiovascular morbidity and disease outcome,^56^, ^57^, ^58^ and add ATTR amyloidosis as a risk factor, thus demonstrating the value of performing autopsies on patients with this emergent disease.

## Conflicts of interest

All authors declare that they have no conflicting interests.

## Author contributions

The study was designed by T. Menter and A. Tzankov. The manuscript was written by T. Menter, J. D. Haslbauer, and A. Tzankov, and partially by H. Hopfer and S. Bassetti. Autopsies were performed by T. Menter, J. D. Haslbauer, A. Tzankov, K. D. Mertz, N. Willi, and D. Turek. Histology was performed by T. Menter, J. D. Haslbauer, N. Deigendesch, S. Frank, S. Savic, H. Hopfer, M. Tolnay, G. Cathomas, K. D. Mertz, N. Willi, D. Turek, and A. Tzankov. PCR analysis was performed by R. Nienhold and K. D. Mertz. Electron microscopy analysis was performed by T. Menter and H. Hopfer. H. Pargger, S. Bassetti and J. D. Leuppi took care of the patients and provided clinical data. All authors read and approved the manuscript.

## Supporting information


**Doc. S1.** Supplementary materials and methods: *in‐corpore* autopsy technique.

**Doc. S2.** Supplementary results: links to open‐source scanned slides of cases.

**Table S1.** Clinical details.

**Table S2.** Detailed autopsy findings.
