# Supplementary material for: Postmortem examination of COVID‐19 patients reveals diffuse alveolar damage with severe capillary congestion and variegated findings in lungs and other organs suggesting vascular dysfunction
Source: Histopathology. 2020 Jul 5;77(2):198–209. doi: 10.1111/his.14134 (PMC7496150; doi:10.1111/his.14134)
Supplement: Supplementary file 1 — Doc. S1. Supplementary materials and methods: in‐corpore autopsy technique. [file HIS-77-198-s001.docx]

**Supplementary methods: *In-corpore* autopsy technique protocol – Institute of Pathology, University Hospital Basel**

Techniques of evisceration vary between pathology institutes. In routine, non-infectious autopsies we typically utilise Ghon’s technique, which involves the removal of thoracic and cervical organs, abdominal organs and urogenital system in separate organ blocks. This allows a thorough topographical inspection of any pertinent anatomy as well as subsequent inspection and weighing of individual organs.

In the light of the current COVID-19 pandemic we attempted to adapt our protocol, limiting the scope of en-bloc organ removal. In this modified *in-corpore* technique detailed below the majority of structures are measured and dissected *in situ*, thus keeping the extent of aerosol generation and exposure to body fluids to a minimum. Furthermore, we have observed a considerably shorter duration of post-mortem examination when employing this technique (45-75 minutes), which may be welcome when performing several infectious autopsies in a limited timeframe. We hope you may find this protocol instructive when encountered with SARS-CoV-2 positive post-mortem examinations at your institute.

- 1. *Preparatory steps before autopsy*

Our mortuary is equipped with a separate dissection room for infectious autopsies, in which all SARS-CoV-2 positive cases were examined. This room allows for adequate airflow (>6 air changes per hour of total room volume). All post-mortem analyses were performed by a resident, an attending pathologist and a trained pathology technician. Adequate protective gear (hazmat suits, boots, goggles, visors, FFP2/3 masks) was provided.

We recommend instilling generous amounts of 4% buffered formalin into the mouth, pharynx and nasal cavity two hours before commencement of autopsy.

- 1. *Autopsy technique*

**External examination and thoracic cavity**

After external examination, perform an I-incision and subsequently remove the breast plate, exposing the thoracic cavity. Record any outstanding findings of the pleura and mediastinum. Then, open the pericardial sac, remove the heart and dissect it *ex corpore* in the direction of blood flow. Inspect lumina of pulmonary arteries to detect any central embolisms. Record the characteristics and amount of pleural effusion, if present. Exenterate cervical organs, trachea and lungs in one organ block (see section *1.3. Dissection of cervical organs, trachea and lung* below).

**Abdomen**

After mobilising and measuring liver dimensions, dissect and check patency of portal structures. Proceed to slice the lobes of the liver *in situ*, allowing inspection of the parenchyma. Open the omental bursa and mobilise the stomach, enabling direct access to the pancreas. Perform an incision at the head of the pancreas parallel to the descending part of the duodenum. Locate the pancreatic duct and open it longitudinally. Measure the dimensions of the spleen and slice it open, examining its parenchyma.

Record the characteristics and amount of ascites, if present. Remove any intestinal adhesions which may be present, taking care to keep intestines intact. Proceed to examine the external surface of small intestines and colon, taking note of any outstanding pathologies. The intestines can then be mobilised cranially to the left or right upper quadrant in order to facilitate the examination of retroperitoneal structures.

**Retroperitoneum**

Locate and slice into the adrenal gland and note any outstanding findings. Open the renal fascia and mobilise the kidney, carefully removing it from its capsule. After recording dimensions and any pertinent findings, perform a longitudinal incision, revealing cortex, medulla and pelvis. Using a probe, locate the ureter and cut along its length until reaching the bladder, checking for patency. Repeat for the contralateral kidney.

**Pelvic cavity**

In female decedents, externally inspect ovaries and uterus, slicing the latter open and recording any outstanding pathologies in the myometrium and uterine cavity.

Next, open the bladder and inspect its contents.

In male patients, a small scalpel should be utilised to dissect the prostate. As its anatomical location can pose a challenge to examine *in situ*, this can be performed together with an autopsy technician.

**Greater vessels**

Using a small scalpel, incise the aorta at L3/4 level. Cut open as far as possible cranially towards the diaphragm and caudally towards the iliac arteries, checking for plaques and other abnormalities.

**Final steps**

Finally, cut open the stomach along the greater curvature, continuing the incision until reaching the end of the duodenum. Inspect the mucosa and note characteristics of stomach contents. Ideally, this should be performed at the end of autopsy to reduce the amount of body fluids in the situs.

Histological analysis is routinely performed for the liver, kidneys, heart, and other regions of specific interest, if applicable. If indicated, a handsaw should be utilised when extracting bone marrow or the brain to reduce aerosol generation.

- 1. *Dissection of cervical organs, trachea and lung*

As described above, we recommend whole organ block exenteration of cervical organs, trachea and lung. Upon removal, the specimen is soaked in a designated container filled with refrigerated (4°C) 4% buffered formalin. Perfuse the lungs with formalin via the trachea, securing them with a clamp when fully expanded. Fixate the specimen in formalin for 72 hours before dissection. Record any outstanding findings of the cervical organs, if applicable. Slice the lungs into 1-1.5cm parasagittal slices and note any outstanding findings. At our institute, we have been using this technique for decades as it has been proven being easier to follow vessels’ lumina and obtain slices of even thickness utilizing sectioning boards with elevated borders, which also minimises the self-cutting risks. We acknowledge that applying parasagittal sections, to some extend hampers comparison of macroscopy with radiological images.
