# Supplementary material for: Postmortem examination of COVID‐19 patients reveals diffuse alveolar damage with severe capillary congestion and variegated findings in lungs and other organs suggesting vascular dysfunction
Source: Histopathology. 2020 Jul 5;77(2):198–209. doi: 10.1111/his.14134 (PMC7496150; doi:10.1111/his.14134)
Supplement: Supplementary file 2 — Doc. S2. Supplementary results: links to open‐source scanned slides of cases. [file HIS-77-198-s002.pdf]

Case	Nr	Legend of sections	
1	A	Liver	https://ictvslidewp01.usb.ch/OlyViaWeb/Html5Viewer?dbId=798403c7-6d3e-4d97-a412-5bc0acd1705f&recordId=42976&layerId=10001&x=206&y=0&scale=3.968496062007751&angle=0&vw=1540&vh=868
	B	Myocardium (posterior wall)	https://ictvslidewp01.usb.ch/OlyViaWeb/Html5Viewer?dbId=798403c7-6d3e-4d97-a412-5bc0acd1705f&recordId=42981&layerId=10001&x=377&y=0&scale=1.7536599032443907&angle=0&vw=1540&vh=868
	C	Kidney (PAS)	https://ictvslidewp01.usb.ch/OlyViaWeb/Html5Viewer?dbId=798403c7-6d3e-4d97-a412-5bc0acd1705f&recordId=42986&layerId=10001&x=68&y=0&scale=4.697694576281936&angle=0&vw=1540&vh=868
	D	Lung, upper lobe (right)	https://ictvslidewp01.usb.ch/OlyViaWeb/Html5Viewer?dbId=798403c7-6d3e-4d97-a412-5bc0acd1705f&recordId=42991&layerId=10001&x=222&y=0&scale=3.552912019826518&angle=0&vw=1540&vh=868
	E	Lung, lower lobe (right)	https://ictvslidewp01.usb.ch/OlyViaWeb/Html5Viewer?dbId=798403c7-6d3e-4d97-a412-5bc0acd1705f&recordId=42996&layerId=10001&x=176&y=0&scale=3.677387857775277&angle=0&vw=1540&vh=868
	F	Lung, upper lobe (left)	https://ictvslidewp01.usb.ch/OlyViaWeb/Html5Viewer?dbId=798403c7-6d3e-4d97-a412-5bc0acd1705f&recordId=43001&layerId=10001&x=174&y=0&scale=3.643679951463077&angle=0&vw=1540&vh=868
	G	Lung, lower lobe (left)	https://ictvslidewp01.usb.ch/OlyViaWeb/Html5Viewer?dbId=798403c7-6d3e-4d97-a412-5bc0acd1705f&recordId=43006&layerId=10001&x=257&y=0&scale=3.462909861613265&angle=0&vw=1540&vh=868
2	A	Liver	https://ictvslidewp01.usb.ch/OlyViaWeb/Html5Viewer?dbId=798403c7-6d3e-4d97-a412-5bc0acd1705f&recordId=43011&layerId=10001&x=105&y=0&scale=4.44914304020503&angle=0&vw=1540&vh=868
	B	Liver (Copper stain)	https://ictvslidewp01.usb.ch/OlyViaWeb/Html5Viewer?dbId=798403c7-6d3e-4d97-a412-5bc0acd1705f&recordId=43016&layerId=10001&x=85&y=0&scale=4.569971310168952&angle=0&vw=1540&vh=868
	C	Myocardium (posterior wall)	https://ictvslidewp01.usb.ch/OlyViaWeb/Html5Viewer?dbId=798403c7-6d3e-4d97-a412-5bc0acd1705f&recordId=43021&layerId=10001&x=158&y=0&scale=3.8601903967757627&angle=0&vw=1540&vh=868
	D	Kidney (PAS)	https://ictvslidewp01.usb.ch/OlyViaWeb/Html5Viewer?dbId=798403c7-6d3e-4d97-a412-5bc0acd1705f&recordId=43026&layerId=10001&x=243&y=0&scale=3.760628215542919&angle=0&vw=1540&vh=868
	E	Lung, upper lobe (right)	https://ictvslidewp01.usb.ch/OlyViaWeb/Html5Viewer?dbId=798403c7-6d3e-4d97-a412-5bc0acd1705f&recordId=43031&layerId=10001&x=283&y=0&scale=2.0846201474754675&angle=0&vw=1540&vh=868
	F	Lung, lower lobe (right)	https://ictvslidewp01.usb.ch/OlyViaWeb/Html5Viewer?dbId=798403c7-6d3e-4d97-a412-5bc0acd1705f&recordId=43036&layerId=10001&x=262&y=0&scale=3.25665376949232&angle=0&vw=1540&vh=868
	G	Lung, upper lobe (left)	https://ictvslidewp01.usb.ch/OlyViaWeb/Html5Viewer?dbId=798403c7-6d3e-4d97-a412-5bc0acd1705f&recordId=43041&layerId=10001&x=185&y=0&scale=3.8398451675335674&angle=0&vw=1540&vh=868

	H	Lung, lower lobe (left)	https://ictvslidewp01.usb.ch/OlyViaWeb/Html5Viewer?dbId=798403c7-6d3e-4d97-a412-5bc0acd1705f&recordId=43046&layerId=10001&x=175&y=0&scale=3.6538053621198543&angle=0&vw=1540&vh=868
3	A	Liver	https://ictvslidewp01.usb.ch/OlyViaWeb/Html5Viewer?dbId=798403c7-6d3e-4d97-a412-5bc0acd1705f&recordId=43051&layerId=10001&x=268&y=0&scale=2.3437684583579443&angle=0&vw=1540&vh=868
	B	Myocardium (anterior wall)	https://ictvslidewp01.usb.ch/OlyViaWeb/Html5Viewer?dbId=798403c7-6d3e-4d97-a412-5bc0acd1705f&recordId=43056&layerId=10001&x=272&y=0&scale=3.389435470339694&angle=0&vw=1540&vh=868
	C	Kidney (PAS)	https://ictvslidewp01.usb.ch/OlyViaWeb/Html5Viewer?dbId=798403c7-6d3e-4d97-a412-5bc0acd1705f&recordId=43061&layerId=10001&x=278&y=0&scale=1.9647732899483625&angle=0&vw=1540&vh=868
	D	Lung, middle lobe (right)	https://ictvslidewp01.usb.ch/OlyViaWeb/Html5Viewer?dbId=798403c7-6d3e-4d97-a412-5bc0acd1705f&recordId=43066&layerId=10001&x=294&y=0&scale=1.933504459430063&angle=0&vw=1540&vh=868
	E	Lung, lower lobe (right)	https://ictvslidewp01.usb.ch/OlyViaWeb/Html5Viewer?dbId=798403c7-6d3e-4d97-a412-5bc0acd1705f&recordId=43071&layerId=10001&x=178&y=0&scale=4.532082398531512&angle=0&vw=1540&vh=868
	F	Lung, upper lobe (left)	https://ictvslidewp01.usb.ch/OlyViaWeb/Html5Viewer?dbId=798403c7-6d3e-4d97-a412-5bc0acd1705f&recordId=43076&layerId=10001&x=247&y=0&scale=3.9366125446218984&angle=0&vw=1540&vh=868
	G	Lung, lower lobe (left)	https://ictvslidewp01.usb.ch/OlyViaWeb/Html5Viewer?dbId=798403c7-6d3e-4d97-a412-5bc0acd1705f&recordId=43081&layerId=10001&x=351&y=0&scale=1.7621430440678502&angle=0&vw=1540&vh=868
	H	Trachea with paratracheal lymph node	https://ictvslidewp01.usb.ch/OlyViaWeb/Html5Viewer?dbId=798403c7-6d3e-4d97-a412-5bc0acd1705f&recordId=43086&layerId=10001&x=354.0232586948169&y=119.12629864885991&scale=1.8100909286665067&angle=0&vw=1540&vh=868
	I	Spleen (PAS)	https://ictvslidewp01.usb.ch/OlyViaWeb/Html5Viewer?dbId=798403c7-6d3e-4d97-a412-5bc0acd1705f&recordId=43091&layerId=10001&x=451&y=0&scale=1.8485292160255557&angle=0&vw=1540&vh=868
4	A	Myocardium (anterior wall)	https://ictvslidewp01.usb.ch/OlyViaWeb/Html5Viewer?dbId=798403c7-6d3e-4d97-a412-5bc0acd1705f&recordId=43096&layerId=10001&x=139&y=0&scale=2.539723177854228&angle=0&vw=1540&vh=868
	B	Lung, upper lobe (right)	https://ictvslidewp01.usb.ch/OlyViaWeb/Html5Viewer?dbId=798403c7-6d3e-4d97-a412-5bc0acd1705f&recordId=43101&layerId=10001&x=243.13194534000036&y=3.7954978399973242&scale=3.8367206778243527&angle=0&vw=1540&vh=868
	C	Lung, middle lobe (right)	https://ictvslidewp01.usb.ch/OlyViaWeb/Html5Viewer?dbId=798403c7-6d3e-4d97-a412-5bc0acd1705f&recordId=43106&layerId=10001&x=178&y=0&scale=3.853362466618111&angle=0&vw=1540&vh=868
	D	Lung, upper lobe (left)	https://ictvslidewp01.usb.ch/OlyViaWeb/Html5Viewer?dbId=798403c7-6d3e-4d97-a412-5bc0acd1705f&recordId=43111&layerId=10001&x=278&y=0&scale=3.5586874009064555&angle=0&vw=1540&vh=868

	E	Lung, lower lobe (left)	https://ictvslidewp01.usb.ch/OlyViaWeb/Html5Viewer?dbId=798403c7-6d3e-4d97-a412-5bc0acd1705f&recordId=43116&layerId=10001&x=173&y=0&scale=4.351729270298852&angle=0&vw=1540&vh=868
	F	Trachea with paratracheal lymph node	https://ictvslidewp01.usb.ch/OlyViaWeb/Html5Viewer?dbId=798403c7-6d3e-4d97-a412-5bc0acd1705f&recordId=43121&layerId=10001&x=349.5273999308849&y=57.458605382255655&scale=4.0412569067504735&angle=0&vw=1540&vh=868
5	A	Liver	https://ictvslidewp01.usb.ch/OlyViaWeb/Html5Viewer?dbId=798403c7-6d3e-4d97-a412-5bc0acd1705f&recordId=43126&layerId=10001&x=163&y=0&scale=4.440962506994963&angle=0&vw=1540&vh=868
	B	Myocardium (posterior wall)	https://ictvslidewp01.usb.ch/OlyViaWeb/Html5Viewer?dbId=798403c7-6d3e-4d97-a412-5bc0acd1705f&recordId=43131&layerId=10001&x=209&y=0&scale=3.7365035858717492&angle=0&vw=1540&vh=868
	C	Kidney (PAS)	https://ictvslidewp01.usb.ch/OlyViaWeb/Html5Viewer?dbId=798403c7-6d3e-4d97-a412-5bc0acd1705f&recordId=43136&layerId=10001&x=233&y=0&scale=3.42493391595188&angle=0&vw=1540&vh=868
	D	Lung, upper lobe (right)	https://ictvslidewp01.usb.ch/OlyViaWeb/Html5Viewer?dbId=798403c7-6d3e-4d97-a412-5bc0acd1705f&recordId=43141&layerId=10001&x=193&y=0&scale=4.0836174181514115&angle=0&vw=1540&vh=868
	E	Lung, middle lobe (right)	https://ictvslidewp01.usb.ch/OlyViaWeb/Html5Viewer?dbId=798403c7-6d3e-4d97-a412-5bc0acd1705f&recordId=43146&layerId=10001&x=172&y=0&scale=4.637836032726666&angle=0&vw=1540&vh=868
	F	Lung, upper lobe (right)	https://ictvslidewp01.usb.ch/OlyViaWeb/Html5Viewer?dbId=798403c7-6d3e-4d97-a412-5bc0acd1705f&recordId=43151&layerId=10001&x=319&y=0&scale=2.1046808994298054&angle=0&vw=1540&vh=868
	G	Lung, upper lobe (left)	https://ictvslidewp01.usb.ch/OlyViaWeb/Html5Viewer?dbId=798403c7-6d3e-4d97-a412-5bc0acd1705f&recordId=43156&layerId=10001&x=360&y=0&scale=1.909856636985595&angle=0&vw=1540&vh=868
	H	Lung, lower lobe (left)	https://ictvslidewp01.usb.ch/OlyViaWeb/Html5Viewer?dbId=798403c7-6d3e-4d97-a412-5bc0acd1705f&recordId=43161&layerId=10001&x=371&y=0&scale=2.358921007654009&angle=0&vw=1540&vh=868
	I	Trachea with paratracheal lymph node	https://ictvslidewp01.usb.ch/OlyViaWeb/Html5Viewer?dbId=798403c7-6d3e-4d97-a412-5bc0acd1705f&recordId=43166&layerId=10001&x=462.204627636165&y=49.88387918542708&scale=2.760891057689126&angle=0&vw=1540&vh=868
6	A	Liver	https://ictvslidewp01.usb.ch/OlyViaWeb/Html5Viewer?dbId=798403c7-6d3e-4d97-a412-5bc0acd1705f&recordId=43171&layerId=10001&x=147&y=0&scale=4.334497220325758&angle=0&vw=1540&vh=868
	B	Myocardium (anterior wall)	https://ictvslidewp01.usb.ch/OlyViaWeb/Html5Viewer?dbId=798403c7-6d3e-4d97-a412-5bc0acd1705f&recordId=43176&layerId=10001&x=253&y=0&scale=3.393110188126069&angle=0&vw=1540&vh=868
	C	Kidney (PAS)	https://ictvslidewp01.usb.ch/OlyViaWeb/Html5Viewer?dbId=798403c7-6d3e-4d97-a412-5bc0acd1705f&recordId=43181&layerId=10001&x=409.72902435175785&y=94.73038311198581&scale=1.6085591931233376&angle=0&vw=1540&vh=868

	D	Lung, upper lobe (right)	https://ictvslidewp01.usb.ch/OlyViaWeb/Html5Viewer?dbId=798403c7-6d3e-4d97-a412-5bc0acd1705f&recordId=43186&layerId=10001&x=237&y=0&scale=2.2200375654397954&angle=0&vw=1540&vh=868
	E	Lung, middle lobe (right)	https://ictvslidewp01.usb.ch/OlyViaWeb/Html5Viewer?dbId=798403c7-6d3e-4d97-a412-5bc0acd1705f&recordId=43191&layerId=10001&x=0&y=136&scale=5.160749816734737&angle=0&vw=1540&vh=868
	F	Lung, upper lobe (left)	https://ictvslidewp01.usb.ch/OlyViaWeb/Html5Viewer?dbId=798403c7-6d3e-4d97-a412-5bc0acd1705f&recordId=43196&layerId=10001&x=323&y=0&scale=1.7722197409557838&angle=0&vw=1540&vh=868
	G	Lung, lower lobe (left)	https://ictvslidewp01.usb.ch/OlyViaWeb/Html5Viewer?dbId=798403c7-6d3e-4d97-a412-5bc0acd1705f&recordId=43201&layerId=10001&x=310&y=0&scale=2.2897654672107497&angle=0&vw=1540&vh=868
	H	Spleen (PAS)	https://ictvslidewp01.usb.ch/OlyViaWeb/Html5Viewer?dbId=798403c7-6d3e-4d97-a412-5bc0acd1705f&recordId=43206&layerId=10001&x=226&y=0&scale=2.4652798579907915&angle=0&vw=1540&vh=868
	I	Bone marrow	https://ictvslidewp01.usb.ch/OlyViaWeb/Html5Viewer?dbId=798403c7-6d3e-4d97-a412-5bc0acd1705f&recordId=43211&layerId=10001&x=0&y=58&scale=4.326127510150335&angle=0&vw=1540&vh=868
7	A	Liver	https://ictvslidewp01.usb.ch/OlyViaWeb/Html5Viewer?dbId=798403c7-6d3e-4d97-a412-5bc0acd1705f&recordId=43216&layerId=10001&x=289&y=0&scale=2.0224260958205913&angle=0&vw=1540&vh=868
	B	Myocardium (posterior wall)	https://ictvslidewp01.usb.ch/OlyViaWeb/Html5Viewer?dbId=798403c7-6d3e-4d97-a412-5bc0acd1705f&recordId=43221&layerId=10001&x=251&y=0&scale=3.2277735410538546&angle=0&vw=1540&vh=868
	C	Kidney (PAS)	https://ictvslidewp01.usb.ch/OlyViaWeb/Html5Viewer?dbId=798403c7-6d3e-4d97-a412-5bc0acd1705f&recordId=43226&layerId=10001&x=171&y=0&scale=3.785001916279862&angle=0&vw=1540&vh=868
	D	Lung, upper lobe (right)	https://ictvslidewp01.usb.ch/OlyViaWeb/Html5Viewer?dbId=798403c7-6d3e-4d97-a412-5bc0acd1705f&recordId=43231&layerId=10001&x=376&y=0&scale=1.745271756204838&angle=0&vw=1540&vh=868
	E	Lung, middle lobe (right)	https://ictvslidewp01.usb.ch/OlyViaWeb/Html5Viewer?dbId=798403c7-6d3e-4d97-a412-5bc0acd1705f&recordId=43236&layerId=10001&x=327&y=0&scale=2.2613598200746976&angle=0&vw=1540&vh=868
	F	Lung, upper lobe (left)	https://ictvslidewp01.usb.ch/OlyViaWeb/Html5Viewer?dbId=798403c7-6d3e-4d97-a412-5bc0acd1705f&recordId=43241&layerId=10001&x=130&y=0&scale=4.283568998255405&angle=0&vw=1540&vh=868
	G	Lung, lower lobe (left)	https://ictvslidewp01.usb.ch/OlyViaWeb/Html5Viewer?dbId=798403c7-6d3e-4d97-a412-5bc0acd1705f&recordId=43246&layerId=10001&x=307&y=0&scale=2.1629661355163385&angle=0&vw=1540&vh=868
	H	Bone marrow	https://ictvslidewp01.usb.ch/OlyViaWeb/Html5Viewer?dbId=798403c7-6d3e-4d97-a412-5bc0acd1705f&recordId=43251&layerId=10001&x=188&y=0&scale=2.8328765027601066&angle=0&vw=1540&vh=868
8	A	Liver	https://ictvslidewp01.usb.ch/OlyViaWeb/Html5Viewer?dbId=798403c7-6d3e-4d97-a412-5bc0acd1705f&recordId=43256&layerId=10001&x=275&y=0&scale=1.9579349904397704&angle=0&vw=1540&vh=868

	B	Myocardium (anterior wall)	https://ictvslidewp01.usb.ch/OlyViaWeb/Html5Viewer?dbId=798403c7-6d3e-4d97-a412-5bc0acd1705f&recordId=43261&layerId=10001&x=265&y=0&scale=3.6058093306288032&angle=0&vw=1540&vh=868
	C	Aortic aneurysm	https://ictvslidewp01.usb.ch/OlyViaWeb/Html5Viewer?dbId=798403c7-6d3e-4d97-a412-5bc0acd1705f&recordId=43266&layerId=10001&x=214&y=0&scale=4.0684023581969315&angle=0&vw=1540&vh=868
	D	Kidney (PAS)	https://ictvslidewp01.usb.ch/OlyViaWeb/Html5Viewer?dbId=798403c7-6d3e-4d97-a412-5bc0acd1705f&recordId=43271&layerId=10001&x=276&y=0&scale=1.9300951983878813&angle=0&vw=1540&vh=868
	E	Lung, upper lobe (right)	https://ictvslidewp01.usb.ch/OlyViaWeb/Html5Viewer?dbId=798403c7-6d3e-4d97-a412-5bc0acd1705f&recordId=43276&layerId=10001&x=360&y=0&scale=2.0380071905495636&angle=0&vw=1540&vh=868
	F	Lung, upper lobe (right)	https://ictvslidewp01.usb.ch/OlyViaWeb/Html5Viewer?dbId=798403c7-6d3e-4d97-a412-5bc0acd1705f&recordId=43281&layerId=10001&x=0&y=106&scale=3.047913566168654&angle=0&vw=1540&vh=868
	G	Lung, lower lobe (left)	https://ictvslidewp01.usb.ch/OlyViaWeb/Html5Viewer?dbId=798403c7-6d3e-4d97-a412-5bc0acd1705f&recordId=43286&layerId=10001&x=378&y=0&scale=1.9438048917036985&angle=0&vw=1540&vh=868
	H	Lung, lower lobe (left)	https://ictvslidewp01.usb.ch/OlyViaWeb/Html5Viewer?dbId=798403c7-6d3e-4d97-a412-5bc0acd1705f&recordId=43291&layerId=10001&x=314&y=0&scale=2.137601970140065&angle=0&vw=1540&vh=868
9	A	Liver	https://ictvslidewp01.usb.ch/OlyViaWeb/Html5Viewer?dbId=798403c7-6d3e-4d97-a412-5bc0acd1705f&recordId=43296&layerId=10001&x=320&y=0&scale=1.909856636985595&angle=0&vw=1540&vh=868
	B	Myocardium (anterior wall)	https://ictvslidewp01.usb.ch/OlyViaWeb/Html5Viewer?dbId=798403c7-6d3e-4d97-a412-5bc0acd1705f&recordId=43301&layerId=10001&x=308&y=0&scale=3.58787722215943&angle=0&vw=1540&vh=868
	C	Myocardium (anterior wall) (Congo red)	https://ictvslidewp01.usb.ch/OlyViaWeb/Html5Viewer?dbId=798403c7-6d3e-4d97-a412-5bc0acd1705f&recordId=43306&layerId=10001&x=385.90266974409166&y=64.4406509866451&scale=2.9101623012047297&angle=0&vw=1540&vh=868
	D	Kidney (PAS)	https://ictvslidewp01.usb.ch/OlyViaWeb/Html5Viewer?dbId=798403c7-6d3e-4d97-a412-5bc0acd1705f&recordId=43311&layerId=10001&x=352&y=0&scale=2.0304279096117472&angle=0&vw=1540&vh=868
	E	Lung, upper lobe (right)	https://ictvslidewp01.usb.ch/OlyViaWeb/Html5Viewer?dbId=798403c7-6d3e-4d97-a412-5bc0acd1705f&recordId=43316&layerId=10001&x=303&y=0&scale=1.8485292160255557&angle=0&vw=1540&vh=868
	F	Trachea + Lung, middle lobe (right)	https://ictvslidewp01.usb.ch/OlyViaWeb/Html5Viewer?dbId=798403c7-6d3e-4d97-a412-5bc0acd1705f&recordId=43321&layerId=10001&x=257&y=0&scale=3.4142511427803175&angle=0&vw=1540&vh=868
	G	Lung, lower lobe (left)	https://ictvslidewp01.usb.ch/OlyViaWeb/Html5Viewer?dbId=798403c7-6d3e-4d97-a412-5bc0acd1705f&recordId=43326&layerId=10001&x=332&y=0&scale=2.0007203054094935&angle=0&vw=1540&vh=868

	H	Lung, lower lobe (left)	https://ictvslidewp01.usb.ch/OlyViaWeb/Html5Viewer?dbId=798403c7-6d3e-4d97-a412-5bc0acd1705f&recordId=43331&layerId=10001&x=310&y=0&scale=3.5073198064887814&angle=0&vw=1540&vh=868
	I	Bone marrow	https://ictvslidewp01.usb.ch/OlyViaWeb/Html5Viewer?dbId=798403c7-6d3e-4d97-a412-5bc0acd1705f&recordId=43336&layerId=10001&x=180&y=0&scale=3.635215495734256&angle=0&vw=1540&vh=868
10	A	Liver and cystic duct	https://ictvslidewp01.usb.ch/OlyViaWeb/Html5Viewer?dbId=798403c7-6d3e-4d97-a412-5bc0acd1705f&recordId=43341&layerId=10001&x=197&y=0&scale=4.144550448106389&angle=0&vw=1540&vh=868
	B	Myocardium (septum)	https://ictvslidewp01.usb.ch/OlyViaWeb/Html5Viewer?dbId=798403c7-6d3e-4d97-a412-5bc0acd1705f&recordId=43346&layerId=10001&x=166&y=0&scale=3.5903700113103895&angle=0&vw=1540&vh=868
	C	Myocardium (septum) (Congo red)	https://ictvslidewp01.usb.ch/OlyViaWeb/Html5Viewer?dbId=798403c7-6d3e-4d97-a412-5bc0acd1705f&recordId=43351&layerId=10001&x=167&y=0&scale=3.4762366634335597&angle=0&vw=1540&vh=868
	D	Kidney (PAS)	https://ictvslidewp01.usb.ch/OlyViaWeb/Html5Viewer?dbId=798403c7-6d3e-4d97-a412-5bc0acd1705f&recordId=43356&layerId=10001&x=292&y=0&scale=2.1933255026601257&angle=0&vw=1540&vh=868
	E	Lung, middle lobe (right)	https://ictvslidewp01.usb.ch/OlyViaWeb/Html5Viewer?dbId=798403c7-6d3e-4d97-a412-5bc0acd1705f&recordId=43361&layerId=10001&x=50&y=0&scale=5.200219983384233&angle=0&vw=1540&vh=868
	F	Lung, lower lobe (right)	https://ictvslidewp01.usb.ch/OlyViaWeb/Html5Viewer?dbId=798403c7-6d3e-4d97-a412-5bc0acd1705f&recordId=43366&layerId=10001&x=423&y=0&scale=1.8330363624364811&angle=0&vw=1540&vh=868
	G	Lung, upper lobe (left)	https://ictvslidewp01.usb.ch/OlyViaWeb/Html5Viewer?dbId=798403c7-6d3e-4d97-a412-5bc0acd1705f&recordId=43371&layerId=10001&x=351&y=0&scale=1.767834838299057 1&angle=0&vw=1540&vh=868
	H	Lung, lower lobe (left)	https://ictvslidewp01.usb.ch/OlyViaWeb/Html5Viewer?dbId=798403c7-6d3e-4d97-a412-5bc0acd1705f&recordId=43376&layerId=10001&x=503.6324670929184&y=32.54261062503866&scale=2.044381604860057&angle=0&vw=1540&vh=868
11	A	Liver	https://ictvslidewp01.usb.ch/OlyViaWeb/Html5Viewer?dbId=798403c7-6d3e-4d97-a412-5bc0acd1705f&recordId=43381&layerId=10001&x=227&y=0&scale=2.416276111044659&angle=0&vw=1540&vh=868
	B	Myocardium (anterior wall)	https://ictvslidewp01.usb.ch/OlyViaWeb/Html5Viewer?dbId=798403c7-6d3e-4d97-a412-5bc0acd1705f&recordId=43386&layerId=10001&x=0&y=72&scale=4.647522044607912&angle=0&vw=1540&vh=868
	C	Kidney (PAS)	https://ictvslidewp01.usb.ch/OlyViaWeb/Html5Viewer?dbId=798403c7-6d3e-4d97-a412-5bc0acd1705f&recordId=43391&layerId=10001&x=261&y=0&scale=3.5242860881357005&angle=0&vw=1540&vh=868
	D	Lung, upper lobe (right)	https://ictvslidewp01.usb.ch/OlyViaWeb/Html5Viewer?dbId=798403c7-6d3e-4d97-a412-5bc0acd1705f&recordId=43396&layerId=10001&x=-497.1394262643262&y=-732.7189460789357&scale=5.484281098202725&angle=0&vw=1540&vh=868

	E	Lung, lower lobe (right)	https://ictvslidewp01.usb.ch/OlyViaWeb/Html5Viewer?dbId=798403c7-6d3e-4d97-a412-5bc0acd1705f&recordId=43401&layerId=10001&x=73&y=0&scale=2.9994870548851273&angle=0&vw=1540&vh=868
	F	Lung, lower lobe (left)	https://ictvslidewp01.usb.ch/OlyViaWeb/Html5Viewer?dbId=798403c7-6d3e-4d97-a412-5bc0acd1705f&recordId=43406&layerId=10001&x=325&y=0&scale=2.0649382027692593&angle=0&vw=1540&vh=868
	G	Lung, lower lobe (left)	https://ictvslidewp01.usb.ch/OlyViaWeb/Html5Viewer?dbId=798403c7-6d3e-4d97-a412-5bc0acd1705f&recordId=43411&layerId=10001&x=168.3466653500002&y=-413.2478663200002&scale=4.219239299978566&angle=0&vw=1540&vh=868
	H	Spleen	https://ictvslidewp01.usb.ch/OlyViaWeb/Html5Viewer?dbId=798403c7-6d3e-4d97-a412-5bc0acd1705f&recordId=43416&layerId=10001&x=338&y=0&scale=2.587816041086797&angle=0&vw=1540&vh=868
	I	Bone marrow	https://ictvslidewp01.usb.ch/OlyViaWeb/Html5Viewer?dbId=798403c7-6d3e-4d97-a412-5bc0acd1705f&recordId=43421&layerId=10001&x=195&y=0&scale=4.473686329776525&angle=0&vw=1540&vh=868
	J	Skin	https://ictvslidewp01.usb.ch/OlyViaWeb/Html5Viewer?dbId=798403c7-6d3e-4d97-a412-5bc0acd1705f&recordId=43426&layerId=10001&x=76&y=0&scale=5.225042619481512&angle=0&vw=1540&vh=868
12	A	Liver	https://ictvslidewp01.usb.ch/OlyViaWeb/Html5Viewer?dbId=798403c7-6d3e-4d97-a412-5bc0acd1705f&recordId=43431&layerId=10001&x=94.57590000000084&y=22.338599999998678&scale=4.647193171730967&angle=0&vw=1540&vh=868
	B	Myocardium (left anterior + posterior wall)	https://ictvslidewp01.usb.ch/OlyViaWeb/Html5Viewer?dbId=798403c7-6d3e-4d97-a412-5bc0acd1705f&recordId=43436&layerId=10001&x=447.51005398404567&y=180.59241022206868&scale=1.503547994116891&angle=0&vw=1540&vh=868
	C	Kidney (PAS)	https://ictvslidewp01.usb.ch/OlyViaWeb/Html5Viewer?dbId=798403c7-6d3e-4d97-a412-5bc0acd1705f&recordId=43441&layerId=10001&x=156&y=0&scale=2.5575544122555622&angle=0&vw=1540&vh=868
	D	Lung, upper and lower lobe (right)	https://ictvslidewp01.usb.ch/OlyViaWeb/Html5Viewer?dbId=798403c7-6d3e-4d97-a412-5bc0acd1705f&recordId=43772&layerId=10001&x=425.48230100000114&y=-117.45545100000061&scale=2.2815089960260777&angle=0&vw=1540&vh=868
	E	Lung, upper and lower lobe (left)	https://ictvslidewp01.usb.ch/OlyViaWeb/Html5Viewer?dbId=798403c7-6d3e-4d97-a412-5bc0acd1705f&recordId=43451&layerId=10001&x=128&y=0&scale=4.465460245370417&angle=0&vw=1540&vh=868
	F	Lung, upper lobe (left)	https://ictvslidewp01.usb.ch/OlyViaWeb/Html5Viewer?dbId=798403c7-6d3e-4d97-a412-5bc0acd1705f&recordId=43456&layerId=10001&x=256&y=0&scale=2.1292653245048343&angle=0&vw=1540&vh=868
	G	Lung, lower lobe (left)	https://ictvslidewp01.usb.ch/OlyViaWeb/Html5Viewer?dbId=798403c7-6d3e-4d97-a412-5bc0acd1705f&recordId=43461&layerId=10001&x=274&y=0&scale=2.38006897881365&angle=0&vw=1540&vh=868

13	A	Liver	https://ictvslidewp01.usb.ch/OlyViaWeb/Html5Viewer?dbId=798403c7-6d3e-4d97-a412-5bc0acd1705f&recordId=43466&layerId=10001&x=157&y=0&scale=2.549982212735681&angle=0&vw=1540&vh=868
	B	Myocardium (left anterior + posterior wall)	https://ictvslidewp01.usb.ch/OlyViaWeb/Html5Viewer?dbId=798403c7-6d3e-4d97-a412-5bc0acd1705f&recordId=43471&layerId=10001&x=155&y=0&scale=4.608546866736491&angle=0&vw=1540&vh=868
	C	Kidney (PAS)	https://ictvslidewp01.usb.ch/OlyViaWeb/Html5Viewer?dbId=798403c7-6d3e-4d97-a412-5bc0acd1705f&recordId=43477&layerId=10001&x=130&y=0&scale=4.434669806613846&angle=0&vw=1540&vh=868
	D	Lung, upper and lower lobe (right)	https://ictvslidewp01.usb.ch/OlyViaWeb/Html5Viewer?dbId=798403c7-6d3e-4d97-a412-5bc0acd1705f&recordId=43482&layerId=10001&x=210&y=0&scale=4.181833579554543&angle=0&vw=1540&vh=868
	E	Lung, lower lobe (right)	https://ictvslidewp01.usb.ch/OlyViaWeb/Html5Viewer?dbId=798403c7-6d3e-4d97-a412-5bc0acd1705f&recordId=43487&layerId=10001&x=210&y=0&scale=3.744184675007372&angle=0&vw=1540&vh=868
	F	Lung, middle lobe (right)	https://ictvslidewp01.usb.ch/OlyViaWeb/Html5Viewer?dbId=798403c7-6d3e-4d97-a412-5bc0acd1705f&recordId=43492&layerId=10001&x=8&y=0&scale=5.438742917283664&angle=0&vw=1540&vh=868
	G	Lung, upper and lower lobe (left)	https://ictvslidewp01.usb.ch/OlyViaWeb/Html5Viewer?dbId=798403c7-6d3e-4d97-a412-5bc0acd1705f&recordId=43497&layerId=10001&x=264.74655766741887&y=-72.08710279408399&scale=2.1402720451841213&angle=0&vw=1540&vh=868
14	A	Myocardium	https://ictvslidewp01.usb.ch/OlyViaWeb/Html5Viewer?dbId=798403c7-6d3e-4d97-a412-5bc0acd1705f&recordId=43502&layerId=10001&x=204&y=0&scale=2.533844189016603&angle=0&vw=1540&vh=868
	B	Lung, upper lobe (right)	https://ictvslidewp01.usb.ch/OlyViaWeb/Html5Viewer?dbId=798403c7-6d3e-4d97-a412-5bc0acd1705f&recordId=43507&layerId=10001&x=31&y=0&scale=4.768412017167382&angle=0&vw=1540&vh=868
	C	Lung, lower lobe (right)	https://ictvslidewp01.usb.ch/OlyViaWeb/Html5Viewer?dbId=798403c7-6d3e-4d97-a412-5bc0acd1705f&recordId=43512&layerId=10001&x=313&y=0&scale=1.8921786520202665&angle=0&vw=1540&vh=868
	D	Lung, lower lobe (right)	https://ictvslidewp01.usb.ch/OlyViaWeb/Html5Viewer?dbId=798403c7-6d3e-4d97-a412-5bc0acd1705f&recordId=43517&layerId=10001&x=224&y=0&scale=3.950820983758123&angle=0&vw=1540&vh=868
	E	Lung, upper lobe (left)	https://ictvslidewp01.usb.ch/OlyViaWeb/Html5Viewer?dbId=798403c7-6d3e-4d97-a412-5bc0acd1705f&recordId=43522&layerId=10001&x=267&y=0&scale=2.3691572842033435&angle=0&vw=1540&vh=868
15	A	Myocardium	https://ictvslidewp01.usb.ch/OlyViaWeb/Html5Viewer?dbId=798403c7-6d3e-4d97-a412-5bc0acd1705f&recordId=43527&layerId=10001&x=107&y=0&scale=4.150084978428553&angle=0&vw=1540&vh=868
	A	Myocardium (Congo red)	https://ictvslidewp01.usb.ch/OlyViaWeb/Html5Viewer?dbId=798403c7-6d3e-4d97-a412-5bc0acd1705f&recordId=43532&layerId=10001&x=156&y=0&scale=3.681744374855022&angle=0&vw=1540&vh=868

	B	Lung, upper lobe (right)	https://ictvslidewp01.usb.ch/OlyViaWeb/Html5Viewer?dbId=798403c7-6d3e-4d97-a412-5bc0acd1705f&recordId=43537&layerId=10001&x=164&y=0&scale=2.389847278984728&angle=0&vw=1540&vh=868
	C	Lung, lower lobe (right)	https://ictvslidewp01.usb.ch/OlyViaWeb/Html5Viewer?dbId=798403c7-6d3e-4d97-a412-5bc0acd1705f&recordId=43542&layerId=10001&x=0&y=0&scale=3.2345508844475072&angle=0&vw=1540&vh=868
	D	Lung, upper lobe (left)	https://ictvslidewp01.usb.ch/OlyViaWeb/Html5Viewer?dbId=798403c7-6d3e-4d97-a412-5bc0acd1705f&recordId=43547&layerId=10001&x=300&y=0&scale=2.1977291608972584&angle=0&vw=1540&vh=868
	E	Lung, lower lobe (left)	https://ictvslidewp01.usb.ch/OlyViaWeb/Html5Viewer?dbId=798403c7-6d3e-4d97-a412-5bc0acd1705f&recordId=43552&layerId=10001&x=0&y=44&scale=5.568243610657967&angle=0&vw=1540&vh=868
16	A	Liver	https://ictvslidewp01.usb.ch/OlyViaWeb/Html5Viewer?dbId=798403c7-6d3e-4d97-a412-5bc0acd1705f&recordId=43557&layerId=10001&x=269&y=0&scale=2.2755089501495105&angle=0&vw=1540&vh=868
	B	Myocardium, anterior and posterior wall (left)	https://ictvslidewp01.usb.ch/OlyViaWeb/Html5Viewer?dbId=798403c7-6d3e-4d97-a412-5bc0acd1705f&recordId=43562&layerId=10001&x=197&y=0&scale=4.087974759228427&angle=0&vw=1540&vh=868
	C	Kidney (PAS)	https://ictvslidewp01.usb.ch/OlyViaWeb/Html5Viewer?dbId=798403c7-6d3e-4d97-a412-5bc0acd1705f&recordId=43777&layerId=10001&x=149&y=0&scale=4.249328297557011&angle=0&vw=1540&vh=868
	D	Lung, upper and lower lobe (left)	https://ictvslidewp01.usb.ch/OlyViaWeb/Html5Viewer?dbId=798403c7-6d3e-4d97-a412-5bc0acd1705f&recordId=43572&layerId=10001&x=148&y=0&scale=4.7076968708290074&angle=0&vw=1540&vh=868
	E	Lung, upper and lower lobe (right)	https://ictvslidewp01.usb.ch/OlyViaWeb/Html5Viewer?dbId=798403c7-6d3e-4d97-a412-5bc0acd1705f&recordId=43577&layerId=10001&x=75&y=0&scale=4.657569850552306&angle=0&vw=1540&vh=868
	F	Lung, lower lobe (right)	https://ictvslidewp01.usb.ch/OlyViaWeb/Html5Viewer?dbId=798403c7-6d3e-4d97-a412-5bc0acd1705f&recordId=43582&layerId=10001&x=370&y=0&scale=2.150198852365424&angle=0&vw=1540&vh=868
	G	Lung, upper lobe (right)	https://ictvslidewp01.usb.ch/OlyViaWeb/Html5Viewer?dbId=798403c7-6d3e-4d97-a412-5bc0acd1705f&recordId=43587&layerId=10001&x=290&y=0&scale=2.0610501516514707&angle=0&vw=1540&vh=868
	H	Spleen	https://ictvslidewp01.usb.ch/OlyViaWeb/Html5Viewer?dbId=798403c7-6d3e-4d97-a412-5bc0acd1705f&recordId=43592&layerId=10001&x=315&y=0&scale=1.989791715170944&angle=0&vw=1540&vh=868
17	A	Liver	https://ictvslidewp01.usb.ch/OlyViaWeb/Html5Viewer?dbId=798403c7-6d3e-4d97-a412-5bc0acd1705f&recordId=43597&layerId=10001&x=186&y=0&scale=2.644105712823808&angle=0&vw=1540&vh=868
	B	Myocardium, anterior and	https://ictvslidewp01.usb.ch/OlyViaWeb/Html5Viewer?dbId=798403c7-6d3e-4d97-a412-5bc0acd1705f&recordId=43602&layerId=10001&x=86&y=0&scale=4.106407946407947&angle=0&vw=1540&vh=868

		posterior wall (left)	
	C	Kidney (PAS)	https://ictvslidewp01.usb.ch/OlyViaWeb/Html5Viewer?dbId=798403c7-6d3e-4d97-a412-5bc0acd1705f&recordId=43607&layerId=10001&x=244&y=0&scale=2.3846409752851913&angle=0&vw=1540&vh=868
	D	Lung, upper and lower lobe (right)	https://ictvslidewp01.usb.ch/OlyViaWeb/Html5Viewer?dbId=798403c7-6d3e-4d97-a412-5bc0acd1705f&recordId=43612&layerId=10001&x=190&y=0&scale=4.1221003033029415&angle=0&vw=1540&vh=868
	E	Lung, upper lobe (right)	https://ictvslidewp01.usb.ch/OlyViaWeb/Html5Viewer?dbId=798403c7-6d3e-4d97-a412-5bc0acd1705f&recordId=43617&layerId=10001&x=193&y=0&scale=2.256697743383503&angle=0&vw=1540&vh=868
	F	Lung, lower lobe (right)	https://ictvslidewp01.usb.ch/OlyViaWeb/Html5Viewer?dbId=798403c7-6d3e-4d97-a412-5bc0acd1705f&recordId=43622&layerId=10001&x=86&y=0&scale=2.7163795948803835&angle=0&vw=1540&vh=868
	G	Lung, lupper and lower lobe (left)	https://ictvslidewp01.usb.ch/OlyViaWeb/Html5Viewer?dbId=798403c7-6d3e-4d97-a412-5bc0acd1705f&recordId=43627&layerId=10001&x=0&y=13&scale=4.975390595421389&angle=0&vw=1540&vh=868
	H	Thrombus in aorta	https://ictvslidewp01.usb.ch/OlyViaWeb/Html5Viewer?dbId=798403c7-6d3e-4d97-a412-5bc0acd1705f&recordId=43632&layerId=10001&x=0&y=32&scale=4.646672992038282&angle=0&vw=1540&vh=868
18	A	Myocardium (left)	https://ictvslidewp01.usb.ch/OlyViaWeb/Html5Viewer?dbId=798403c7-6d3e-4d97-a412-5bc0acd1705f&recordId=43637&layerId=10001&x=173&y=0&scale=4.000468084723335&angle=0&vw=1540&vh=868
	B	Lung, upper lobe (right)	https://ictvslidewp01.usb.ch/OlyViaWeb/Html5Viewer?dbId=798403c7-6d3e-4d97-a412-5bc0acd1705f&recordId=43642&layerId=10001&x=180&y=0&scale=2.3358106190411116&angle=0&vw=1540&vh=868
	C	Lung, lower lobe (right)	https://ictvslidewp01.usb.ch/OlyViaWeb/Html5Viewer?dbId=798403c7-6d3e-4d97-a412-5bc0acd1705f&recordId=43647&layerId=10001&x=31&y=0&scale=5.27546829372522&angle=0&vw=1540&vh=868
	D	Lung, upper lobe (left)	https://ictvslidewp01.usb.ch/OlyViaWeb/Html5Viewer?dbId=798403c7-6d3e-4d97-a412-5bc0acd1705f&recordId=43652&layerId=10001&x=161&y=0&scale=2.389950094648081&angle=0&vw=1540&vh=868
	E	Lung, lower lobe (left)	https://ictvslidewp01.usb.ch/OlyViaWeb/Html5Viewer?dbId=798403c7-6d3e-4d97-a412-5bc0acd1705f&recordId=43657&layerId=10001&x=277&y=0&scale=2.268887141733972&angle=0&vw=1540&vh=868
19	A	Liver	https://ictvslidewp01.usb.ch/OlyViaWeb/Html5Viewer?dbId=798403c7-6d3e-4d97-a412-5bc0acd1705f&recordId=43662&layerId=10001&x=35&y=0&scale=4.494862043854681&angle=0&vw=1540&vh=868
	B	Myocardium, anterior and posterior wall (left)	https://ictvslidewp01.usb.ch/OlyViaWeb/Html5Viewer?dbId=798403c7-6d3e-4d97-a412-5bc0acd1705f&recordId=43667&layerId=10001&x=0&y=28&scale=3.9363576909444004&angle=0&vw=1540&vh=868

	C	Kidney (PAS)	https://ictvslidewp01.usb.ch/OlyViaWeb/Html5Viewer?dbId=798403c7-6d3e-4d97-a412-5bc0acd1705f&recordId=43672&layerId=10001&x=178&y=0&scale=3.1281921333445956&angle=0&vw=1540&vh=868
	D	Lung, upper and lower lobe (right)	https://ictvslidewp01.usb.ch/OlyViaWeb/Html5Viewer?dbId=798403c7-6d3e-4d97-a412-5bc0acd1705f&recordId=43677&layerId=10001&x=115&y=0&scale=4.078071519678464&angle=0&vw=1540&vh=868
	E	Lung, upper lobe (right)	https://ictvslidewp01.usb.ch/OlyViaWeb/Html5Viewer?dbId=798403c7-6d3e-4d97-a412-5bc0acd1705f&recordId=43682&layerId=10001&x=263&y=0&scale=2.487300894366276&angle=0&vw=1540&vh=868
	F	Lung, lower lobe (right)	https://ictvslidewp01.usb.ch/OlyViaWeb/Html5Viewer?dbId=798403c7-6d3e-4d97-a412-5bc0acd1705f&recordId=43687&layerId=10001&x=260&y=0&scale=2.171548076265306&angle=0&vw=1540&vh=868
	G	Lung, upper and lower lobe (left)	https://ictvslidewp01.usb.ch/OlyViaWeb/Html5Viewer?dbId=798403c7-6d3e-4d97-a412-5bc0acd1705f&recordId=43692&layerId=10001&x=362&y=0&scale=1.940562585693451&angle=0&vw=1540&vh=868
20	A	Liver	https://ictvslidewp01.usb.ch/OlyViaWeb/Html5Viewer?dbId=798403c7-6d3e-4d97-a412-5bc0acd1705f&recordId=43697&layerId=10001&x=300.67525092698224&y=38.331757187023555&scale=3.51932670905211&angle=0&vw=1540&vh=868
	B	Myocardium, anterior and posterior wall (left)	https://ictvslidewp01.usb.ch/OlyViaWeb/Html5Viewer?dbId=798403c7-6d3e-4d97-a412-5bc0acd1705f&recordId=43702&layerId=10001&x=140&y=0&scale=3.8120791552654376&angle=0&vw=1540&vh=868
	C	Kidney (PAS)	https://ictvslidewp01.usb.ch/OlyViaWeb/Html5Viewer?dbId=798403c7-6d3e-4d97-a412-5bc0acd1705f&recordId=43782&layerId=10001&x=218&y=0&scale=2.328784925276153&angle=0&vw=1540&vh=868
	D	Lung, upper and lower lobe (right)	https://ictvslidewp01.usb.ch/OlyViaWeb/Html5Viewer?dbId=798403c7-6d3e-4d97-a412-5bc0acd1705f&recordId=43712&layerId=10001&x=116&y=0&scale=3.4794209525002544&angle=0&vw=1540&vh=868
	E	Lung, lower lobe (right)	https://ictvslidewp01.usb.ch/OlyViaWeb/Html5Viewer?dbId=798403c7-6d3e-4d97-a412-5bc0acd1705f&recordId=43717&layerId=10001&x=324&y=0&scale=1.8285413340794258&angle=0&vw=1540&vh=868
	F	Lung, upper and lower lobe (left)	https://ictvslidewp01.usb.ch/OlyViaWeb/Html5Viewer?dbId=798403c7-6d3e-4d97-a412-5bc0acd1705f&recordId=43722&layerId=10001&x=206&y=0&scale=3.6165489404641775&angle=0&vw=1540&vh=868
	G	Lung, lower lobe (left)	https://ictvslidewp01.usb.ch/OlyViaWeb/Html5Viewer?dbId=798403c7-6d3e-4d97-a412-5bc0acd1705f&recordId=43727&layerId=10001&x=111&y=0&scale=4.306231408001706&angle=0&vw=1540&vh=868
21	A	Liver	https://ictvslidewp01.usb.ch/OlyViaWeb/Html5Viewer?dbId=798403c7-6d3e-4d97-a412-5bc0acd1705f&recordId=43732&layerId=10001&x=277&y=0&scale=3.7403401870102763&angle=0&vw=1540&vh=868

	B	Myocardium, posterior wall	https://ictvslidewp01.usb.ch/OlyViaWeb/Html5Viewer?dbId=798403c7-6d3e-4d97-a412-5bc0acd1705f&recordId=43737&layerId=10001&x=0&y=46&scale=5.79687982472908&angle=0&vw=1540&vh=868
	C	Myocardium, posterior wall (Congo red)	https://ictvslidewp01.usb.ch/OlyViaWeb/Html5Viewer?dbId=798403c7-6d3e-4d97-a412-5bc0acd1705f&recordId=43742&layerId=10001&x=116&y=0&scale=4.411383421180628&angle=0&vw=1540&vh=868
	D	Kidney (PAS)	https://ictvslidewp01.usb.ch/OlyViaWeb/Html5Viewer?dbId=798403c7-6d3e-4d97-a412-5bc0acd1705f&recordId=43747&layerId=10001&x=231&y=0&scale=2.1689197762832965&angle=0&vw=1540&vh=868
	E	Lung, upper and lower lobe (right)	https://ictvslidewp01.usb.ch/OlyViaWeb/Html5Viewer?dbId=798403c7-6d3e-4d97-a412-5bc0acd1705f&recordId=43752&layerId=10001&x=250&y=0&scale=3.887609783407397&angle=0&vw=1540&vh=868
	F	Lung, upper lobe (right)	https://ictvslidewp01.usb.ch/OlyViaWeb/Html5Viewer?dbId=798403c7-6d3e-4d97-a412-5bc0acd1705f&recordId=43757&layerId=10001&x=326&y=0&scale=2.069939450395901&angle=0&vw=1540&vh=868
	G	Lung, upper and lower lobe (left)	https://ictvslidewp01.usb.ch/OlyViaWeb/Html5Viewer?dbId=798403c7-6d3e-4d97-a412-5bc0acd1705f&recordId=43762&layerId=10001&x=269&y=0&scale=3.844262791401756&angle=0&vw=1540&vh=868
	H	Lung, lower lobe (left)	https://ictvslidewp01.usb.ch/OlyViaWeb/Html5Viewer?dbId=798403c7-6d3e-4d97-a412-5bc0acd1705f&recordId=43767&layerId=10001&x=291&y=0&scale=1.8867962978687271&angle=0&vw=1540&vh=868
