# Supplementary material for: Postmortem examination of COVID‐19 patients reveals diffuse alveolar damage with severe capillary congestion and variegated findings in lungs and other organs suggesting vascular dysfunction
Source: Histopathology. 2020 Jul 5;77(2):198–209. doi: 10.1111/his.14134 (PMC7496150; doi:10.1111/his.14134)
Supplement: Supplementary file 3 — Table S1. Clinical details. [file HIS-77-198-s003.docx]

| **Case** | **Age** | **Sex** | **BMI** | **Initial presentation** | **Hospitalisation period (days)** | **Period between positive nasal swab PCR and death (days)** | **Radiology findings** | | **Relevant Drug Intake** | | **Comorbidities** | | | | |
| --- | --- | --- | --- | --- | --- | --- | --- | --- | --- | --- | --- | --- | --- | --- | --- |
|  |  |  |  |  |  |  | Ground glass opacities | Consolidation | **Interfering with RAAS*** | **Immuno-suppressant** | **Cardiovascular (with risk factors)** | **Pulmonary (with risk factors)** | **Diabetes** | **Others** |  |
| **1** | 68 | F | 35 | Cough, fever | 9 | 9 | yes | no | yes | no | Hypertension, atherosclerosis, obesity | - | - | Multiple sclerosis |  |
| **2** | 86 | M | 26 | Cough, vertigo, rhinitis | 5 | 5 | no | yes | yes | yes | Hypertension, dyslipidaemia | Sleep apnea | Type II | tMDS, S.p. APML and prostate cancer |  |
| **3** | 96 | M | 23 | Fever, flu-like symptoms | 3 | 3 | yes | yes | yes | no | Hypertension, coronary artery disease, S.p. myocardial infarction |  | - | Parkinson`s disease, dementia |  |
| **4** | 78 | M | 44 | Cough, fever, tachypnea | 3 | 3 | yes | yes | yes | no | Hypertension, dyslipidaemia, obesity, aortic valve reconstruction, atrial fibrillation, coronary artery disease | Sleep apnea, smoker | Type II | Chronic renal failure, hyperuricemia |  |
| **5** | 66 | M | 29 | Cough, fatigue, dyspnea, pre-renal failure | 9 | 9 | yes | yes | yes | no | Hypertension | Smoker | - | - |  |
| **6** | 74 | M | 27 | Cough, fever, dyspnea, pre-renal failure | 3 | 5 | yes | yes | yes | no | Coronary artery disease, hypertension | Smoker, COPD, wedge resection of lung | Type II | Metastatic prostate carcinoma, chronic renal failure, genetic thrombophilia |  |
| **7** | 81 | F | 26 | Dry throat, polydipsia, diarrhoea | 4 | 5 | yes | no | yes | no | Coronary artery disease, peripheral artery disease, hypertension | Smoker | - |  |  |
| **8** | 71 | M | 25 | Septic shock, decubitus | 0 | 0 | yes | yes | no | no | Peripheral arterial disease, infrarenal aortic aneurysm, coronary heart disease, valvulopathy, S.p. double bypass | - | - | - |  |
| **9** | 88 | M | 28 | Cough, dyspnea, ankle oedema, diarrhoea, pancytopenia | 2 | 2 | yes | no | no | yes | Heart failure, coronary and hypertensive heart disease, atrial fibrillation | - | - | Waldenström`s macroglobulinemia, S.p. SCC and basalioma |  |
| **10** | 85 | M | 29 | Tachypnea, dyspnea, acute renal failure | 5 | 5 | yes | no | yes | no | Hypertensive cardiomyopathy, eccentric hypertrophy with hyperdynamic EF, persistent atrial fibrillation | Ex-smoker (40py) | - | - |  |
| **11** | 58 | M | 47 | Cough, dyspnea | 7 | 12 | yes | yes | yes | no | Hypertension, obesity, atherosclerosis | - | - | - |  |
| **12** | 75 | M | 27 | Cough, dyspnea, acute renal failure | 3 | 3 | yes | yes | no | no | Dyslipidaemia, hypertension, coronary artery disease | Sleep apnea | Type II |  |  |
| **13** | 53 | M | 59 | Dyspnea, acute renal failure | 8 | 6 | nil | yes | no | no | Hypertension, obesity | Sleep apnea, S.p. pneumonia-associated ARDS, causing respiratory failure type 1. Patient on long-term oxygen therapy | Type II | Liver cirrhosis, acquired immunosuppression |  |
| **14** | 94 | F | 19 | Dry cough, dyspnea, fever | 0 | unknown | nil | nil | yes | no | Hypertension, tachycardia-bradycardia syndrome, atrial fibrillation, valvulopathy | - | - | Dementia |  |
| **15** | 89 | M | 26 | Cough, dyspnea, fever | 5 | 7 | nil | yes | yes | no | Coronary artery disease, hypertension, valvulopathy, third-degree atrioventricular block, dyslipidaemia | Ex-smoker | Type II | - |  |
| **16** | 61 | F | 41 | Cough, fever | 9 | 9 | yes | yes | no | no | Hypertension, obesity | - | Type II | - |  |
| **17** | 72 | M | 25 | Productive cough, fever | 12 | 14 | no | no | no | no | Hypertension | Sleep apnea, ex-smoker | - | Gout |  |
| **18** | 79 | M | 27 | Somnolence, fever, malaise, cough | 16 | 16 | nil | yes | yes | no | Hypertension, dyslipidaemia, coronary artery disease | - | - | Alzheimer’s, Parkinson’s disease |  |
| **19** | 65 | M | 26 | Cough, fever, acute renal failure | 7 | 20 | yes | yes | no | no | Hypertension, coronary artery disease, S.p. STEMI | Sleep apnea, COPD, ex-smoker (90py) | - | Bipolar disorder |  |
| **20** | 71 | M | 36 | Cough, fever, sore throat | 4 | 9 | nil | yes | no | no | Hypertension, obesity, dyslipidaemia | COPD | - | Gout |  |
| **21** | 96 | M | 25 | Cough, fever, gait uncertainty | 13 | 15 | nil | nil | no | no | Hypertension, coronary artery disease | - | - | Dementia |  |

* Includes all agents interfering with RAAS, directly or indirectly: ACE-inhibitors, AT1-inibitors (ARBs), renin-Inhibitors, aldosterone-antagonists
