# Supplementary material for: Postmortem examination of COVID‐19 patients reveals diffuse alveolar damage with severe capillary congestion and variegated findings in lungs and other organs suggesting vascular dysfunction
Source: Histopathology. 2020 Jul 5;77(2):198–209. doi: 10.1111/his.14134 (PMC7496150; doi:10.1111/his.14134)
Supplement: Supplementary file 4 — Table S2. Detailed autopsy findings. [file HIS-77-198-s004.docx]

| **Case** | **Cause of death** | **Lung and upper airways** | **Cardiovascular** | **Kidney** | **Liver** | **Bone marrow** | **CNS** | **Sequelae of shock** | **Others** |
| --- | --- | --- | --- | --- | --- | --- | --- | --- | --- |
| 1 | SARS-CoV-2 associated respiratory failure with superimposed bacterial bronchopneumonia | Mucostasis, bilateral interstitial pneumonia with lymphocytic infiltrates, reactive pneumocytes, intraalveolar exudates with beginning alveolar damage with capillary congestion, bronchopneumonia in left lower lobe, microthrombi in alveolar capillaries | Generalised atherosclerosis, bilateral pleural effusions, mild myocardial hypertrophy, coronary sclerosis, myocardial infarction scar, heart weight: 360g, 65. percentile | Renal congestion, arteriosclerosis, vascular scarring | Macro- and microvesicular steatosis | Osteoporosis | Demyelinising lesions in frontal, parietal and occipital semioval centre. Loss of myelin sheaths without inflammatory changes, indicative of chronic multiple sclerosis lesions; atrophy of optic nerve |  |  |
| 2 | SARS-CoV-2 associated respiratory failure | Mucostasis, alveolar damage in early exudative phase with capillary congestion, subpleural interstitial oedema, reactive pneumocytes, fibrinous exudate | Generalised atherosclerosis, normal heart weight, stenosis of RIVA and RCA, diffuse myocardial fibrosis, heart weight: 358g, 60. percentile | Acute tubular injury, moderate arteriolosclerosis | Liver cirrhosis with macrovesicular steatosis, portal inflammation and increased copper deposition (NASH/ASH and/or heterozygous Wilson’s disease) | Therapy-related MDS-EB2-F | - | Acute tubular kidney injury , adrenal gland fatigue, peracute myocardial contraction band necroses |  |
| 3 | SARS-CoV-2 associated respiratory failure with superimposed bacterial bronchopneumonia | Mucosal nodularity of trachea due to erythroid extravasation and fibrooedema  Interstitial oedema and severe bacterial bronchopneumonia, Gram-positive cocci in alveolar spaces, capillary congestion, microthrombi in alveolar capillaries | Eccentric myocardial hypertrophy | Acute tubular injury, vascular scarring | Hepatic congestion, periportal microvesicular steatosis, cholestasis | - | Hydrocephalus internus, Parkinson’s disease with Lewy bodies in substantia nigra and locus coeruleus with moderate loss of dopaminergic neurons. Arteriosclerosis of perivascular space of basal ganglia, acute hypoxic ischemic encephalopathy of hippocampus | Acute tubular kidney injury, adrenal gland fatigue | Reactive hilar lymph nodes with sinus ectasia and increased presence of reactive plasmablasts and capillary congestion  Splenic hyperplasia of white pulp, atypical lymphocytes and plasmablasts in sinus |
| 4 * | SARS-CoV-2 associated cardiorespiratory failure | Severe fibrinous erosive tracheitis  Alveolar damage in early exudative phase with capillary congestion, subpleural intraalveolar and interstitial oedema | Cor bovinum maximum (780 g, >95. percentile), peracute posterior myocardial infarction, biological aortic valve implant, severe coronary sclerosis | n/a | n/a | n/a | n/a | n/a | Paratracheal lymph nodes with sinus ectasia and increased presence of plasmablasts |
| 5 | SARS-CoV-2 associated respiratory failure with superimposed bacterial bronchopneumonia leading to multi organ failure | Fibrinous tracheitis  Bilateral pleural effusions, combination of exudative and proliferative alveolar damage with capillary congestion and severe bronchopneumonia with fibrinous exudates and reactive pneumocytes | Eccentric biventricular myocardial hypertrophy (425 g, 85. percentile), fibrosis of papillary muscles, valvular degeneration, coronary sclerosis, biatrial dilation, generalised atherosclerosis | Acute tubular injury, hypertensive nephropathy and arteriosclerosis, vascular scarring | Macrovesicular steatosis | - | - | Perivenular haemorrhagic necroses in the liver, acute tubular kidney injury , contraction band necrosis in myocardium, splenomegaly with reactive changes, haemorrhagic fatty necroses of pancreas | - |
| 6 | SARS-CoV-2 associated respiratory failure with superimposed bacterial bronchopneumonia | Alveolar damage in early exudative phase with capillary congestion, peribronchiolar metaplasia, interstitial haemorrhages, intraalveolar and interstitial oedema and beginning bronchopneumonia. Pre-existing interstitial fibrosis and pleural/mediastinal adhesions | Eccentric myocardial hypertrophy (430 g, 60. percentile), posterior myocardial fibrosis, valvular degeneration, coronary sclerosis and stenosis of RIVA, infrarenal atherosclerosis | Acute tubular injury, vascular scarring, diabetic glomerulopathy, diffuse/nodular glomerulosclerosis and severe arteriolosclerosis, pyelitis, pyelonephritis scar | - | Bone marrow metastases of prostate carcinoma, reactive left shifted myelopoeisis | - | Shock necroses of liver acute tubular kidney injury , adrenal gland fatigue, splenomegaly with reactive changes | Adenocarcinoma of prostate (ypT3a, ypN1, ypM1b, V0, L1, Pn1) |
| 7 | SARS-CoV-2 associated respiratory failure with superimposed bacterial bronchopneumonia | Alveolar damage in early exudative phase with capillary congestion, peribronchiolar metaplasia of left lower lobe, oedema, emphysema, beginning bronchopneumonia | Eccentric myocardial hypertrophy (440g, >95. percentile), dilation of left atrium with organising thrombi, posterior myocardial infarction scar, fibrosis of papillary muscles, sclerosis of coronary arteries with stent in RVA, valvular degeneration, generalised atherosclerosis | Acute tubular injury , arteriolosclerosis, vascular scarring | Macro- and microvesicular steatosis | Left shifted myelopoeisis | - | Hepatic congestion, acute tubular kidney injury | - |
| 8 | SARS-CoV-2 associated respiratory failure with superimposed bacterial bronchopneumonia | Tracheitis and bronchitis  Bilateral bronchopneumonia with alveolar damage in exudative stage, capillary congestion, acute capillaritis and alveolitis, microthrombi in alveolar capillaries | Eccentric myocardial hypertrophy with biatrial dilation (510 g, 95. percentile), apical aneurysm of left ventricle with adherent thrombus, posterolateral myocardial infarction scar, fibrosis of papillary muscles, stenoses of RVA, RIVA, RCX, double bypass, S.p. left atrial appendage resection, aortic valve implant, generalized atherosclerosis, aortic aneurysm with prosthesis, pericardial adhesions | Acute tubular injury , vascular scarring, nephrocalcinosis, arteriolosclerosis | Macro- and microvesicular steatosis (20%) | - | Severe atherosclerotic changes of C. arteriosus, hydrocephalus internus, cerebral oedema | Acute tubular kidney injury, haemorrhagic necrosis of liver | - |
| 9 | SARS-CoV-2 associated respiratory failure | Alveolar damage in exudative and proliferative stage with capillary congestion, acute bronchitis, metaplasia of alveolar epithelium, moderate emphysema, oedema, periphery pulmonary embolism of right middle lobe | Senile ATTR amyloidosis. Eccentric myocardial hypertrophy (480g, 90. percentile) with biatrial dilation, myocardial left-ventricular fibrosis, endocardial fibrosis, coronary sclerosis | Vascular scarring, nephrocalcinosis | - | Diffuse infiltrates of lymphoplasmacytic lymphoma | - |  | Cystic lesion of pancreas (low grade PanIN)  Erosive gastritis |
| 10 | SARS-CoV-2 associated respiratory failure leading to multi organ failure | Alveolar damage in exudative stage with capillary congestion and reactive pneumocytic changes, emphysema, atelectasis of left lower lobe, pulmonary artery sclerosis, microthrombi in alveolar capillaries, presence of amyloid deposits in pulmonary vessels | Senile ATTR amyloidosis. Eccentric myocardial hypertrophy (right > left, 550g, > 95. percentile) with biatrial dilation, coronary sclerosis, atherosclerosis | Moderate interstitial fibrosis and tubular atrophy | Hepatic congestion, macro- and microvesicular steatosis (15%) | - | - | Adrenal gland fatigue | Splenic hyaloserositis |
| 11 | SARS-CoV-2 associated respiratory failure leading to multi organ failure | Alveolar damage in exudative stage with capillary congestion and reactive pneumocytic changes, diffuse pulmonary haemorrhage and central & peripheral pulmonary embolisms, microthrombi in alveolar capillaries | Eccentric myocardial hypertrophy (left > right, 460g, >95. percentile). | Acute tubular injury | Hepatic congestion | 1-5% of histiocytes with haemophagocytic activity, in line with the clinical macrophage activation syndrome | - | Acute tubular kidney injury, adrenal gland fatigue | Mucostatic frontal sinusitis  Haemorrhage of psoas muscle and sternocleidomastoid |
| 12 | SARS-CoV-2 associated respiratory failure with superimposed bacterial bronchopneumonia | Mucopurulent bronchitis, capillary congestion, bronchopneumonia of left lower lobe, mononuclear and lymphocytic interstitial infiltrate, pulmonary haemorrhage, mild emphysema, peribronchiolar metaplasia, anthracosis | Senile ATTR amyloidosis. Eccentric hypertrophy (435g, 80. percentile), coronary sclerosis, infrarenal atherosclerosis | Acute tubular injury, diabetic nephropathy | Macro- and microvesicular steatosis | - | - | Acute tubular kidney injury, reactive changes in spleen | Benign prostatic hyperplasia, diverticulosis of sigmoid colon  Multiple cutaneous haemorrhages, haemorrhage of psoas muscle |
| 13 | SARS-CoV-2 associated respiratory failure | Alveolar damage in exudative and concomitant proliferative phase with capillary congestion, reactive pneumocyte changes, lymphocytic interstitial infiltrate, peribronchiolar metaplasia, residues of central and peripheral pulmonary embolisms, pulmonal artery sclerosis | Eccentric hypertrophy (655g, >95. percentile) coronary sclerosis, patent foramen ovale, atherosclerosis | Acute tubular injury, vascular scarring | Steatohepatitis and micronodular liver cirrhosis (NASH/ASH) | Hypercellular with left shifted myelopoeisis | - | Acute tubular kidney injury, splenomegaly with reactive changes | - |
| 14 * | SARS-CoV-2 associated respiratory failure | Organising bronchopneumonia, capillary congestion, prominent lymphocytic interstitial infiltrate , oedema, presence of amyloid deposits in pulmonary vessels | Senile ATTR amyloidosis | - | - | - | - | - | - |
| 15 * | SARS-CoV-2 associated respiratory failure | Alveolar damage in exudative and concomitant proliferative phase with capillary congestion, reactive pneumocyte changes, lymphocytic interstitial infiltrate, oedema | Senile ATTR amyloidosis. Myocardial fibrosis | - | - | - | - | - | - |
| 16 | SARS-CoV-2 associated respiratory failure | Alveolar damage in exudative and beginning concomitant proliferative phase with capillary congestion, lymphocytic interstitial infiltrate | Eccentric hypertrophy (550g, >95. percentile), coronary sclerosis, myocardial fibrosis, biatrial dilation, generalized atherosclerosis | Acute tubular injury, vascular scarring | Steatohepatitis (NASH/ASH) | Spondylosis | - | Acute tubular kidney injury, reactive changes in spleen | Struma diffusa with lymphofollicular inflammation, in line with Hashimoto’s disease |
| 17 | SARS-CoV-2 associated respiratory failure | Alveolar damage in proliferative phase with prominent interstitial lymphocytic infiltrate and capillary congestion, emphysema, acute pulmonary infarction in right lower lobe | Mild eccentric hypertrophy (450g, 85. percentile), biatrial dilation | Acute tubular injury, renal infarction, thrombotic microangiopathy, arteriosclerosis | - | - | - | Acute tubular kidney injury | Thrombotic microangiopathy in the lung and the kidneys |
| 18 * | SARS-CoV-2 associated respiratory failure | Organising pneumonia with focal bronchopneumonia, giant cells, peribronchiolar metaplasia, capillary congestion | - | - | - | - | - | - | - |
| 19 | SARS-CoV-2 associated respiratory failure | Alveolar damage in exudative phase with capillary congestion, minimal lymphocytic interstitial infiltrate, oedema, emphysema, peribronchiolar metaplasia | Eccentric hypertrophy (485g, > 95. percentile), myocardial infarction scar, coronary sclerosis (stent in RIVA), patent foramen ovale, atherosclerosis | Acute tubular injury, vascular scarring | Macrovesicular steatosis (30%) | - | - | Acute tubular kidney injury | Lipomatosis of pancreas, cholecystolithiasis, diverticulosis, benign prostate hyperplasia |
| 20 | SARS-CoV-2 associated respiratory failure with superimposed bacterial bronchopneumonia | Alveolar damage in exudative and proliferative phase with capillary congestion, reactive pneumocyte changes, lymphocytic interstitial infiltrate, diffuse bronchopneumonia, giant cells, peribronchiolar metaplasia, silicoanthracosis, pulmonary artery sclerosis. | Heart weight 475g, 50. percentile, coronary artery sclerosis, generalised atherosclerosis | Acute tubular injury, thrombotic microangiopathy, vascular scarring | Macrovesicular steatosis (5%), hepatic congestion | - | - | Acute tubular kidney injury | Systemic thrombotic angiopathy in lungs, glomerula of kidneys and left adrenal gland  Benign prostate hyperplasia  Myelolipoma of right adrenal gland |
| 21 | SARS-CoV-2 associated respiratory failure | Alveolar damage in concomitant exudative and proliferative phase with capillary congestion | Senile ATTR amyloidosis. Eccentric hypertrophy (570g, >95. percentile) with biatrial dilation, coronary sclerosis , pacemaker in right ventricle; severe generalised atherosclerosis | Acute tubular injury, thrombotic microangiopathy, diffuse glomerulosclerosis, vascular scarring | Macro- and microvesticular steatosis (40%) | - | - | Acute tubular kidney injury, adrenal gland fatigue, reactive changes in spleen | Adenocarcinoma of prostate (pT3, pN0, pM0, L0, V0, Pn1) |

* Performance of partial autopsy: heart, lungs and upper airways only
